# Supplementary material for: Impact of physical and chemical parameters on spinoculation for chimeric antigen receptor T cell manufacturing using a quality-by-design approach
Source: Mol Ther Adv. 2026 Feb 10;34(1):201691. doi: 10.1016/j.omta.2026.201691 (PMC13148932; doi:10.1016/j.omta.2026.201691)
Supplement: Document S1. Figures S1–S4 and Tables S1–S3 [file mmc1.pdf]

OMTA, Volume *34*

Supplemental information

Impact of physical and chemical parameters on spinoculation for chimeric antigen receptor T cell manufacturing using a quality-by-design approach

Pedro Silva Couto, Dale J. Stibbs, Braulio Carrillo Sanchez, Pierre Springuel, Syd McLean, Ursula Schultz, Manuel Effenberger, Yasuhiro Takeuchi, and Qasim A. Rafiq

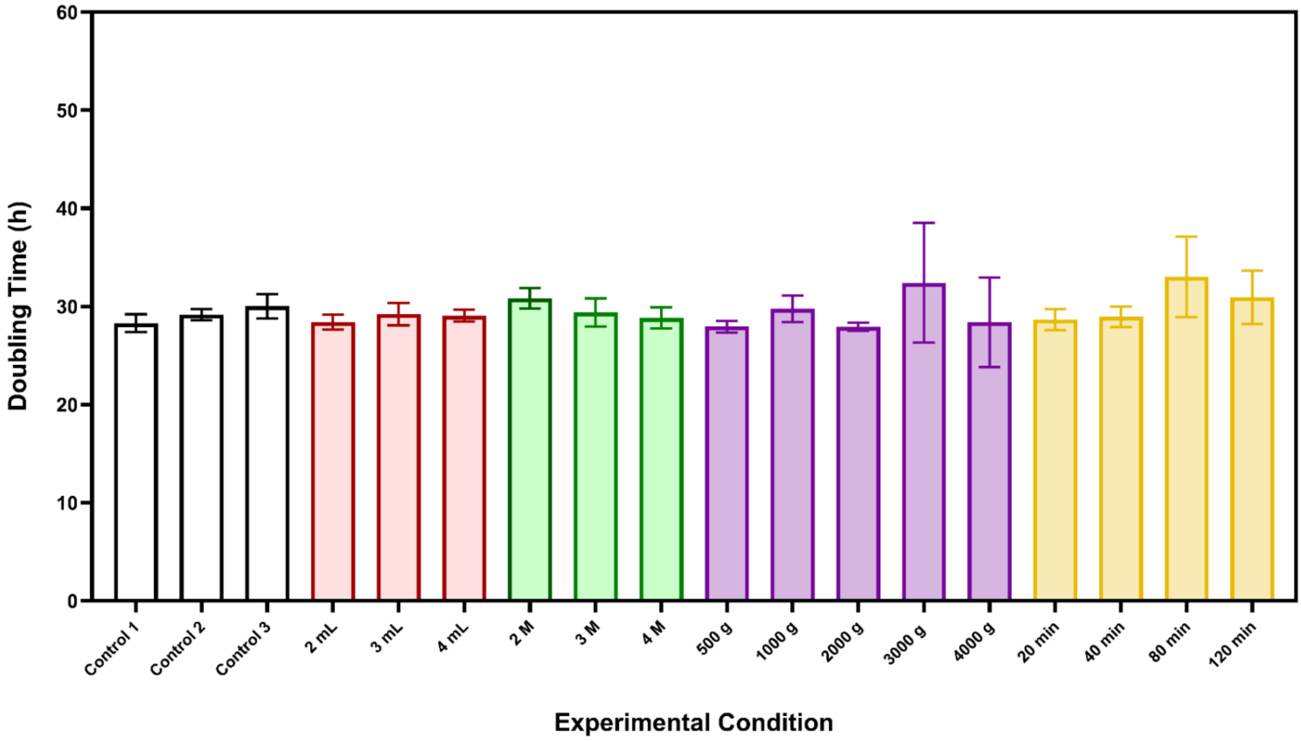

FIGURE S1-Representation of the doubling time across conditions tested in the evaluation of physical parameters on transduction efficiency via spinoculation (N=3). Bars representing mean and standard deviation represented as error bars.

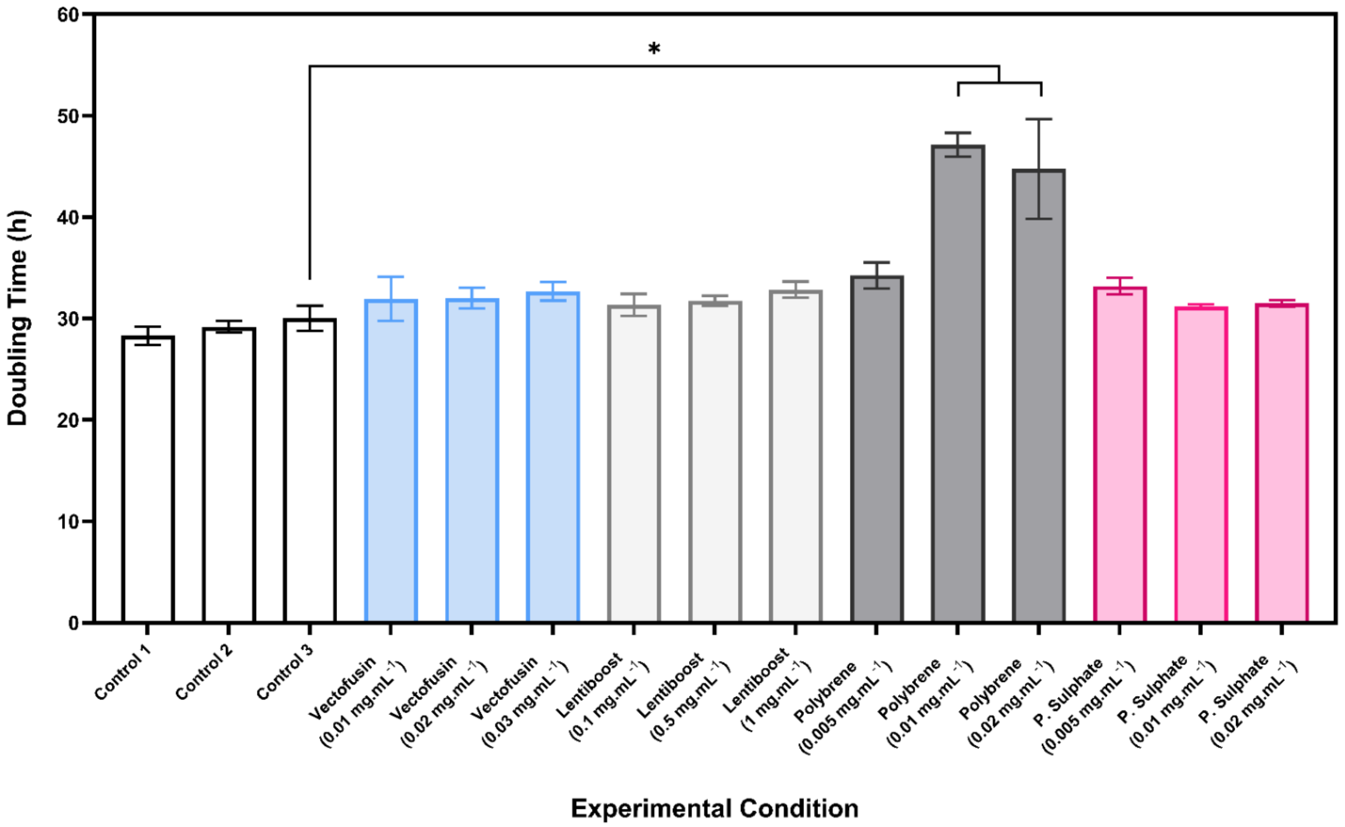

FIGURE S2-Representation of the doubling time across conditions tested in the evaluation of chemical parameters on transduction efficiency via spinoculation (N=3). Bars representing mean and standard deviation represented as error bars.

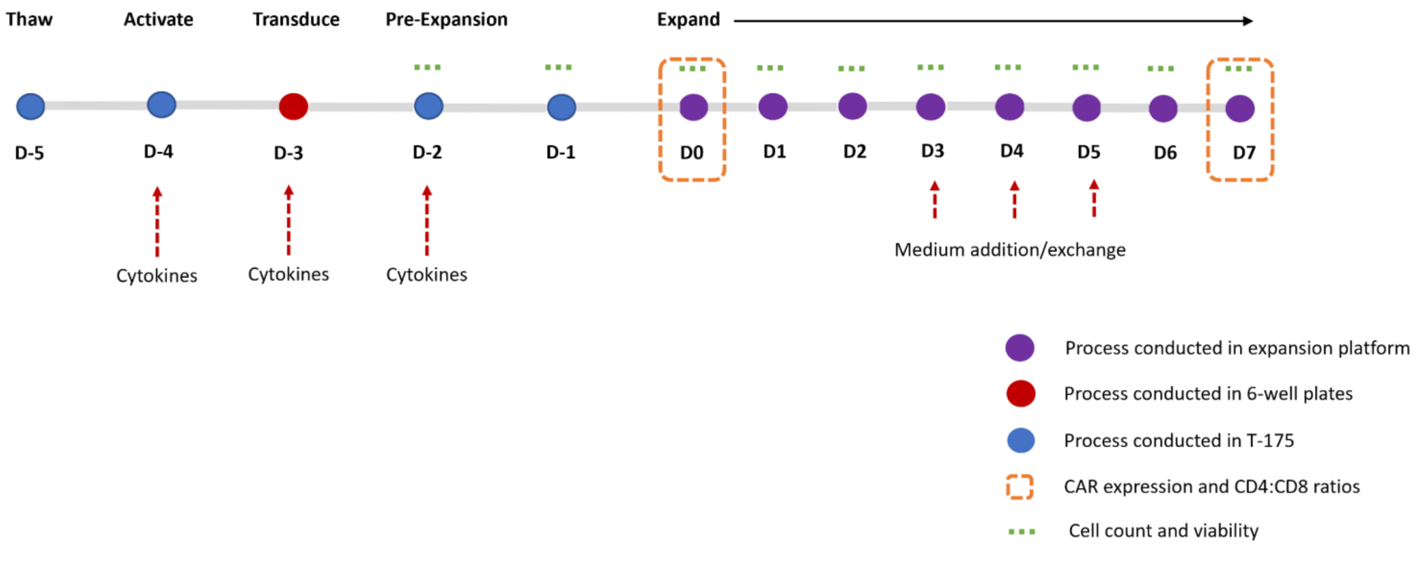

FIGURE S3-Manufacturing process used to compare the baseline process to the optimised process and optimised process when performed with serum/xeno-free medium.

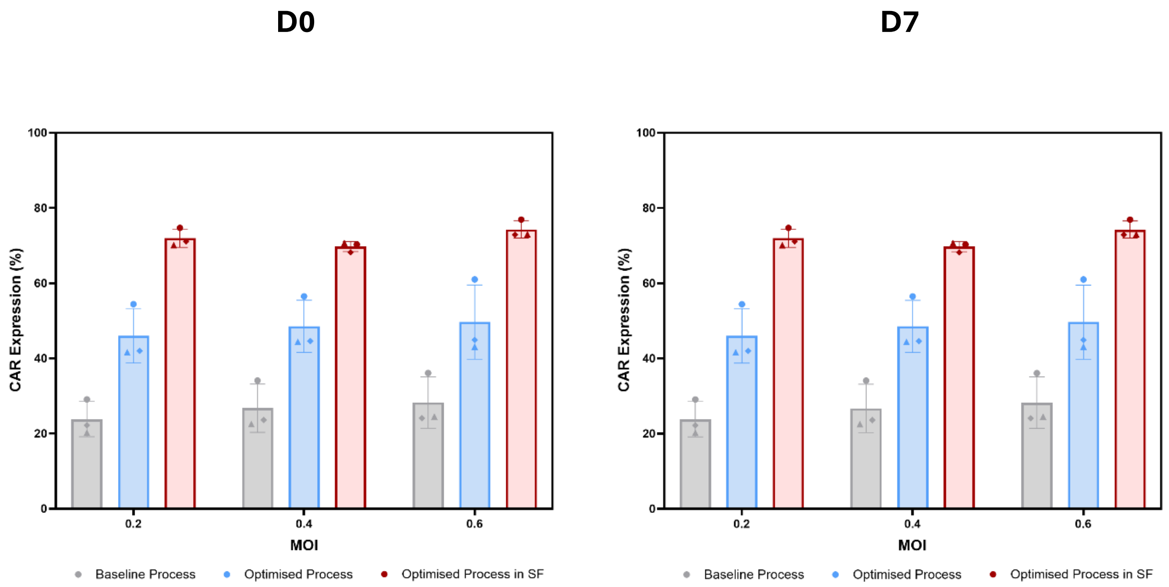

FIGURE S4-Evaluation of the impact of different MOIs across the different transduction processes (N=3). Bars representing mean and standard deviation represented as error bars.

TABLE S1-List of functional titre values obtained across biological donors and titration methods.

Titration Method	Donor	Replicate	Titre (TU.mL^{-1})
Jurkat	1	A	2.37×10^{9}
		B	2.55×10^{9}
		C	2.37×10^{9}
	2	A	2.36×10^{9}
		B	2.59×10^{9}
		C	2.36×10^{9}
	3	A	2.31×10^{9}
		B	2.95×10^{9}
		C	2.31×10^{9}
Jurkat + retronectin	1	A	6.49×10^{9}
		B	7.65×10^{9}
		C	6.49×10^{9}
	2	A	6.66×10^{9}
		B	9.28×10^{9}
		C	7.89×10^{9}
	3	A	6.51×10^{9}
		B	7.98×10^{9}
		C	1.02×10^{10}
T-cells	1	A	2.12×10^{7}
		B	2.02×10^{7}
		C	2.03×10^{7}
	2	A	1.43×10^{7}
		B	1.33×10^{7}
		C	1.35×10^{7}
	3	A	1.58×10^{7}
		B	1.48×10^{7}
		C	1.50×10^{7}
T-cells + retronectin	1	A	2.57×10^{7}
		B	2.43×10^{7}
		C	2.46×10^{7}
	2	A	2.22×10^{7}
		B	2.11×10^{7}
		C	2.13×10^{7}
	3	A	2.34×10^{7}
		B	2.24×10^{7}
		C	2.26×10^{7}

TABLE S2-Summary of model adequacy metrics, including R^2, RMSE, and ANOVA p-values, used to assess the performance of the DoE models.

Response	Significant Factors	RMSE	R^2	ANOVA p-value
Viability (%)	Donor, Lentiboost, Polybrene and Centrifugation Speed	0.762	0.984	< 0.0001
CAR%	Lentiboost, Polybrene, Centrifugation Speed	4.170	0.963	< 0.0001
CD4:CD8 Ratio	Donor, Lentiboost	0.415	0.901	< 0.001
Doubling Time	Donor	0.088	0.912	< 0.001

TABLE S3-List of factors, variables units and levels used in the design of experiment studies used in this manuscript.

Variable List	Factor	Unit	Levels	
**Independent Variables**	T cell Donor	Donor Number	1, 2, 3	
	LentiBOOST™	$mg.mL^{-1}$	0	1
	Polybrene	$mg.mL^{-1}$	0	0.005
	Centrifugation Speed	g	1000	2000
**Dependent Variables**	CAR Expression	%	-	
	CD4:CD8 Ratio	No units	-	
	Doubling Time	hours	-	
	Viability	%	-	
