# Supplementary material for: Impact of physical and chemical parameters on spinoculation for chimeric antigen receptor T cell manufacturing using a quality-by-design approach
Source: Mol Ther Adv. 2026 Feb 10;34(1):201691. doi: 10.1016/j.omta.2026.201691 (PMC13148932; doi:10.1016/j.omta.2026.201691)
Supplement: Document S2. Article plus supplemental information [file mmc2.pdf]

Molecular Therapy
Advances
Original Article

Impact of physical and chemical parameters on spinoculation for chimeric antigen receptor T cell manufacturing using a quality-by-design approach

Pedro Silva Couto,[1,2] Dale J. Stibbs,[2] Braulio Carrillo Sanchez,[2] Pierre Springuel,[2] Syd McLean,[2] Ursula Schultz,[3] Manuel Effenberger,[3] Yasuhiro Takeuchi,[4,5] and Qasim A. Rafiq[2]

[1]Department of Chemical Engineering, University of Bath, Claverton Down, Bath BA2 7AY, UK; [2]Department of Biochemical Engineering, University College London, Bernard Katz Building, Gower Street, London WC1E 6BT, UK; [3]Sartorius CellGenix GmbH, Am Flughafen 16, 79108 Freiburg im Breisgau, Germany; [4]Division of Infection and Immunity, University College London, Cruciform Building, Gower Street, London WC1E 6BT, UK; [5]Biotherapeutics and Advanced Therapies, Scientific Research and Innovation, Medicines, and Healthcare Products Regulatory Agency, South Mimms, London EN6 3QG, UK

Chimeric antigen receptor (CAR) T cell therapies represent a significant advancement for treating hematological malignancies, particularly in relapsed/refractory cases. Despite their clinical success, the high cost of CAR T cell therapies remains a major barrier to broader implementation. A significant proportion of these costs stems from the dependency on viral vectors and the limited understanding of transduction mechanisms. This work evaluates the impact of physical and chemical parameters during transduction using a spinoculation process. Physical parameters, such as spinoculation duration and speed, were identified as key drivers of transduction efficiency, contributing to a 20%–30% increase in transduction. Similarly, the addition of LentiBOOST and polybrene enhanced transduction efficiency by approximately 1–2-fold compared with control conditions without these supplements. Given that both physical and chemical parameters influence transduction efficiency, a quality-by-design approach was used to systematically investigate their potential synergistic or antagonistic interactions. This systematic approach highlighted the cytotoxic impact of polybrene and demonstrated that LentiBOOST is critical to drive transduction, particularly in CD4 subsets. The optimized process led to a 2–3-fold improvement in transduction without compromising CAR T cell growth or functionality and was shown to be compatible with serum- and xeno-free medium, supporting its translational potential.

INTRODUCTION

Gene-modified cell therapies have revolutionized healthcare by engineering human cells with therapeutic genes to restore or enhance their function. Chimeric antigen receptor (CAR) T cell therapies, such as Abecma, Yescarta, and Kymriah, and gene-edited hematopoietic stem cell therapies, such as Casgevy, Lyfgenia, and Lenmeldy, are FDA-approved examples of cell therapies developed to treat a range of diseases, including hematological malignancies, as well as inherited blood disorders.[1–4] Efficient and cost-effective gene delivery remains one of the main manufacturing challenges and a significant contributor to overall production costs.[5–9] In addition, current gene transfer mechanisms are poorly understood, and the use of black-box manufacturing equipment provides limited insight into the process conditions, limiting optimization studies and overall process efficiency.[10–12]

The vast majority of approved gene-modified cell therapies rely on the transfer of a therapeutic gene. From a manufacturing perspective, this is typically achieved using either non-viral delivery methods or viral vectors.[8,13] Although non-viral methods offer a promising alternative, their application is often constrained by low efficiency and a dependence on physical or chemical delivery systems, which can induce significant cytotoxicity.[14–16] In contrast, viral vectors, such as lentiviral vectors (LVs) and gammaretroviral vectors, are widely used for gene knock-in because of their high transduction efficiency and stable gene integration.[17] However, a significant portion of the manufacturing cost is driven by two main factors: the process complexity associated with viral vector production and purification and the limited understanding of how to maximize vector transfer during transduction, both of which contribute to process inefficiencies and variability.

LVs can transduce both dividing and non-dividing cells, whereas gamma-retroviral vectors are largely restricted to actively dividing cells. In addition, LVs can package larger transgenes (around 8–10 kb) compared with retroviral vectors, allowing delivery of more complex genetic payloads.[17,18]

Two major types of factors have been described to modulate transduction *in vitro*: physical and chemical.[19–22] Physical parameters influence the interaction between viral vectors and target cells and

Received 15 October 2025; accepted 5 February 2026;
https://doi.org/10.1016/j.omta.2026.201691.

Correspondence: Pedro Silva Couto, Department of Chemical Engineering, University of Bath, Claverton Down, Bath BA2 7AY, UK.
E-mail: psc66@bath.ac.uk

Published by Elsevier Inc. on behalf of The American Society of Gene and Cell Therapy.
This is an open access article under the CC BY license (http://creativecommons.org/licenses/by/4.0/).

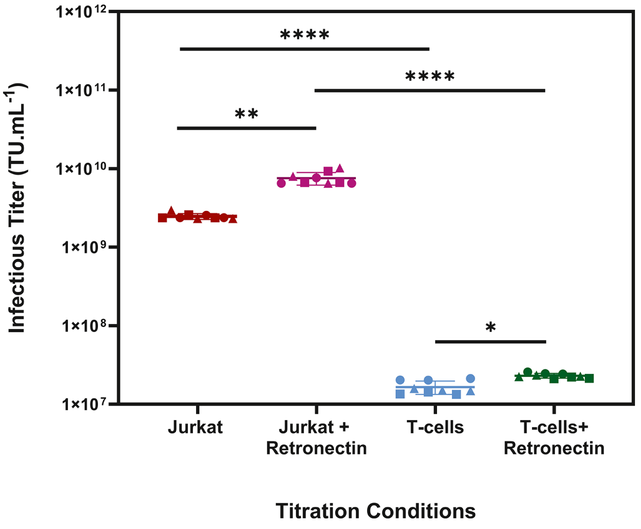

Figure 1. Infectious titer determination

Infectious titer of VSV-G-pseudotyped 3rd generation LVs carrying a CAR transgene, titrated using Jurkat and primary T cells in the presence or absence of retronectin, with a spinoculation protocol (1,000 g, 40 min).

Three independent vials of Jurkat cells and three independent T cell donors were used to account for batch-to-batch and biological variability, respectively, represented by different symbols. The group mean infectious titer across all donors is shown with the corresponding standard deviation ($N = 9$).

include factors such as cell concentration, transduction volume, and multiplicity of infection (MOI), as well as techniques such as spinoculation, a centrifugation-based method that enhances contact between cells and viral particles.[23,24] Although several studies have demonstrated that spinoculation enhances transduction by increasing contact between vector and cells and by inducing cytoskeletal rearrangements, there has been limited research into the specific parameters that influence this process.[21,23,25,26] Factors such as cell concentration, spinoculation volume, centrifugation time, and centrifugal force are likely to play a critical role in determining transduction efficiency.[20,23–25,27] Despite their importance, these variables are often optimized in isolation without considering their combined effects on cell-vector interactions. Such interdependencies can influence not only the proportion of transduced cells but also the kinetics of gene transfer, yet they remain poorly characterized in current manufacturing protocols. Systematic evaluation of these parameters is therefore essential to fully optimize transduction processes.

Chemical parameters involve additives that interact with either the vector particles, the target cells, or both, altering the chemical environment in which transduction occurs.[20,28,29] These can be broadly divided into two groups: polycations, which reduce electrostatic repulsion between the negatively charged cell membranes and viral envelopes by introducing positively charged molecules, and membrane-active compounds, which transiently increase cell membrane permeability to facilitate vector entry. Although several studies have explored the use of chemical agents to enhance transduction, these investigations are typically limited to a narrow set of conditions, often testing only a single concentration or a very restricted range and rarely assessing potential synergistic effects between different enhancers.[19,30–32] Most transduction studies use one-factor-at-a-time approaches, which fail to capture interactions between parameters. As a result, the poor understanding of transduction processes limits the identification of the fundamental factors driving efficient vector entry and integration.

Finally, medium formulation remains an important factor influencing transduction efficiency. While fetal bovine serum (FBS) supplementation has been reported to both enhance and inhibit transduction, its undefined composition reduces process consistency.[33–35] In addition to existing donor-to-donor variability, FBS addition poses a challenge to implementing robust, quality-by-design (QbD) manufacturing strategies.

To address these limitations, a QbD approach that systematically identifies and controls critical process parameters is required, enabling the definition of operating ranges that improve reproducibility, consistency, and overall transduction efficiency.[36–38]

This study aims to evaluate the impact of physical and chemical parameters in spinoculation-based transduction processes used for CAR T cell manufacturing. This was achieved by first evaluating the individual contributions of physical and chemical parameters using a one-factor-at-a-time approach, followed by assessing potential synergies between factors through a design of experiments (DoE) study. The optimized process was then compared with the baseline and further evaluated under serum- and xeno-free medium (SXFM) conditions to assess its suitability for clinical manufacturing.

RESULTS

Impact of titration conditions on infectious titer

The initial stage of this study was designed to quantify the impact of cell type and process conditions on infectious titer determination (Figure 1). This study demonstrated that titrating the LV preparation with Jurkat cells instead of primary T cells can lead to an overestimation of the infectious titer by over 150–300×. Titration with Jurkat cells and primary T cells resulted in infectious titers of 2.4×10^9 and 1.6×10^7 TU.mL^{-1}, respectively. This difference was more accentuated when the comparison was established per cell type using retronectin with Jurkat and primary T cells, resulting in infectious titers of 7.6×10^9 and 2.3×10^7 TU.mL^{-1}, respectively. Furthermore, the use of retronectin was also demonstrated to result in a higher functional titer, irrespective of the cell type used. In light of the differences shown herein, the experiments in this study were conducted using the MOI obtained from titration on retronectin-coated plates with the same three primary T cell donors used throughout the experimental work (Table S1). This approach aimed to minimize experimental variability and to ensure that the calculated MOI was directly relevant to the conditions used for T cell transduction.

Effects of physical and chemical parameters on transduction

Throughout the different sections of this work, an MOI of 0.2 was used with retronectin-coated plates to facilitate the

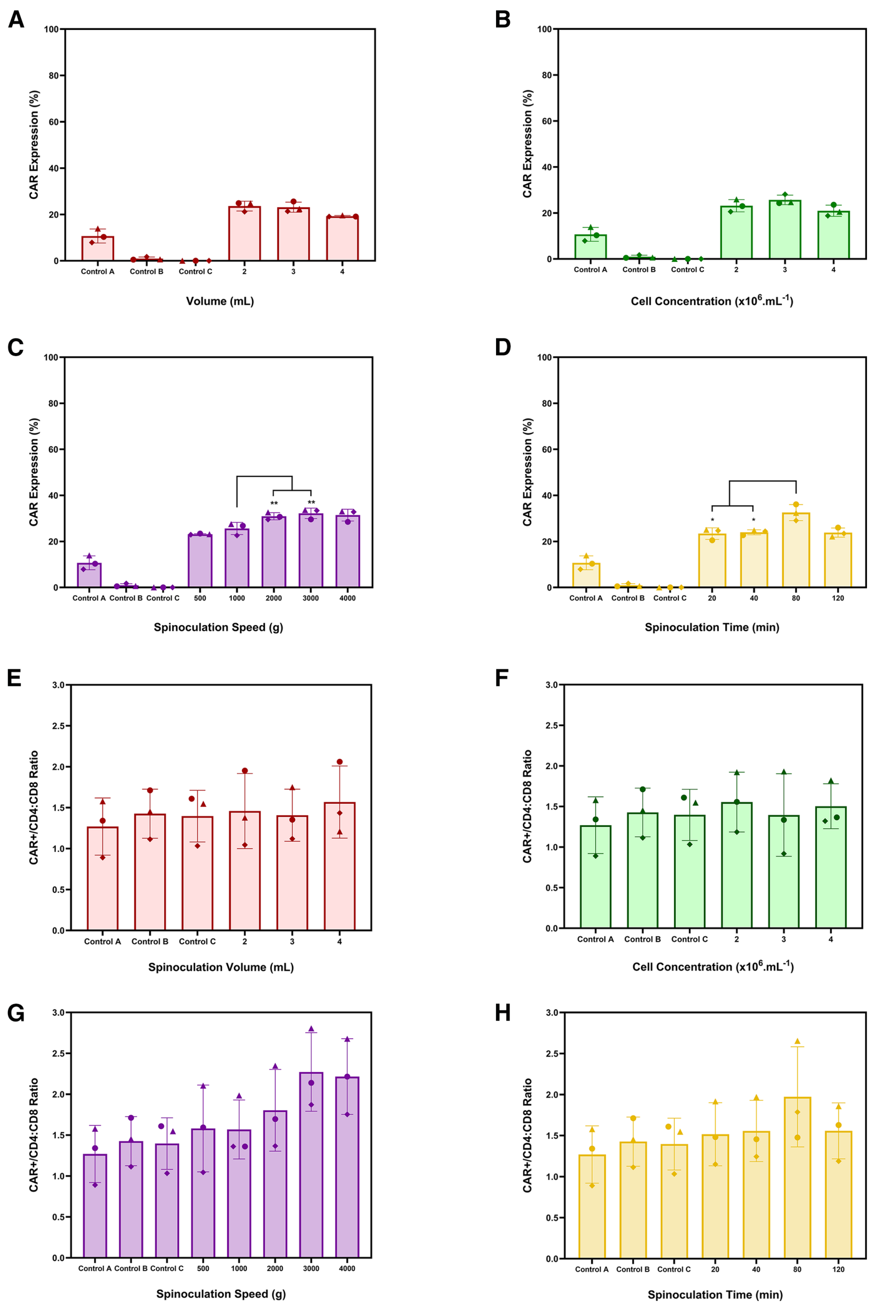

Figure 2. Impact of physical factors on transduction

Representation of CAR transduction efficiency when (A) volume, (B) cell concentration, (C) spinoculation speed, and (D) time were varied at an MOI of 0.2. Impact of (E) volume, (F) cell concentration, (G) spinoculation speed, and (H) time on CAR+/CD4:CD8. Bars represent the mean, and error bars indicate one standard deviation from three biological replicates (*N* = 3). Control A represents LV addition without spinoculation, control B represents spinoculation without LV, and control C lacked both LV and spinoculation. For controls B and C, the CD4:CD8 ratio obtained (marked with #) corresponds to non-transduced cells.

spinoculation process. An assessment was carried out to evaluate how physical parameters, including volume, cell concentration, centrifugation speed, and duration of spinoculation, influence transduction efficiency (Figures 2A–2D) and CD4:CD8 ratios (Figures 2E–2H).

Using the 6-well plate system described in the Methods section, performing spinoculation with reaction volumes between 2 and 4 mL did not significantly affect CAR expression, which remained at an average of 22 ± 2.6% (Figure 2A). A similar trend was observed when concentrations were varied between 2 and 4×10^6 cells.mL^{-1}, with CAR expression averaging 23.2 ± 2.9% (Figure 2B). Increasing the spinoculation speed to 2000 and 3000 g improved transduction efficiencies to 30.9 ± 1.3% and 32.2 ± 1.8%, respectively. No further increase was seen at 4000 g, with efficiencies of 31.4 ± 2.1% (Figure 2C). A spinoculation time of 80 min was demonstrated to improve CAR expression to 32.5 ± 2.8% compared with both shorter (20 and 40 min) and longer cycles (120 min), which averaged 23.7 ± 0.7% (Figure 2D).

Although the volume (Figure 2E) and cell concentration (Figure 2F) at which spinoculation was performed did not lead to changes in the CD4:CD8 (1.5 ± 0.4 and 1.4 ± 0.3, respectively), increasing the spinoculation speed showed a trend toward a higher ratio (Figure 2G). This was evidenced by an increase in the CD4:CD8 ratio from 1.6 ± 0.4 to 2.3 ± 0.4 at 500 and 3000 g, respectively. Increasing spinoculation time showed a similar trend, with longer cycle durations leading to higher CD4:CD8 ratios, increasing from 1.5 ± 0.3 to 1.9 ± 0.2 for the 20- and 80-min cycles, respectively (Figure 2H).

To evaluate the potential impact of physical parameters on cell growth kinetics post-transduction, population doubling time was calculated under the various experimental conditions (Figure S1). The results demonstrated that, within the evaluated design space for these physical parameters, no adverse effects were observed on doubling time, which averaged 29.5 h.

An assessment was then carried out to evaluate how chemical parameters, including Vectofusin-1, protamine sulfate, polybrene, and LentiBOOST, influence transduction efficiency (Figures 3A–3D) and the CD4:CD8 ratio of the CAR+ cells (Figures 3E–3H).

The addition of Vectofusin-1 did not result in any improvements in transduction efficiency, as demonstrated by CAR expression levels of 23.9 ± 0.9% in the absence of the molecule and 22.8 ± 0.8% when the molecule was added at 0.03 $mg.mL^{-1}$ (Figure 3A). Adding LentiBOOST to the spinoculation reactions led to an increase in CAR expression of approximately 2.5×, from 23.9 ± 1.2% in its absence to 57.1 ± 3.0% at a concentration of 1 $mg.mL^{-1}$ (Figure 3B). Supplementing the cultures with 0.005–0.015 $mg.mL^{-1}$ polybrene approximately doubled CAR expression from 23.9 ± 0.9% to 44.1 ± 2.8% (Figure 3C). Protamine sulfate did not significantly improve CAR expression, with levels remaining similar in its absence (23.9 ± 1.2%) and in its presence at the highest tested concentration of 0.02 $mg.mL^{-1}$ (28.9 ± 0.3%) (Figure 3D).

Regarding the CD4:CD8 ratios within the CAR+ fractions, no statistical differences were observed when Vectofusin-1 was added to the culture. These averaged 1.5 ± 0.4 and 1.7 ± 0.3 in the absence and presence of the molecule, respectively (Figure 3E). The addition of LentiBOOST led to an increase in the CD4:CD8 ratio in a dose-dependent manner, with 1 $mg.mL^{-1}$ providing a ratio of 2.5 ± 0.4 compared with 1.5 ± 0.4 in its absence (Figure 3F). Despite its contribution to increasing CAR expression, the addition of polybrene did not cause any changes in the CD4:CD8 ratio of CAR+ fractions (1.5 ± 0.4 and 1.6 ± 0.2) in the absence and at a concentration of 0.015 $mg.mL^{-1}$, respectively (Figure 3G). Finally, the addition of protamine sulfate to the spinoculation process resulted in ratios averaging 1.5 ± 0.4 compared with 1.7 ± 0.2 in its absence (Figure 3H).

Finally, although LentiBOOST, protamine sulfate and Vectofusin-1 did not impact cell growth kinetics, with an average doubling time of 31.5 ± 1.8 h, polybrene at concentrations above 0.005 $mg.mL^{-1}$ had a detrimental impact on the population doubling time, increasing it to an average of 46.9 ± 3.45 h (Figure S2). Altogether, these data suggest a clear improvement in CAR expression levels by increasing centrifugation speed to 2000 g and adding both LentiBOOST and polybrene at concentrations of 1 and 0.005 $mg.mL^{-1}$, respectively.

Interactions between physical and chemical parameters during transduction

To assess whether the physical and chemical parameters that enhance CAR expression have synergistic effects, a DoE approach was employed. In this study, spinoculation speed (1000–2000 g), LentiBOOST concentration (0–1 $mg.mL^{-1}$), and polybrene (0–0.005 $mg.mL^{-1}$), along with three independent biological donors, were included as independent variables in the DoE study. The dependent variables assessed were viability, CAR transduction efficiency, CD4:CD8 ratio, and doubling time (Figures 4A–4F).

The predicted cell viability response from the DoE analysis indicated that donor variability, LentiBOOST, polybrene, and centrifugation speed each had a negative impact on this parameter (Table S2). As illustrated in Figure 4A, polybrene exerted a markedly greater impact on reducing cell viability 1 day post-transduction compared with LentiBOOST. Nonetheless, across the design space examined in this study, all tested transduction conditions resulted in cell viability levels above 90% at harvesting (Figure 4B).

Regarding CAR expression, the model identified LentiBOOST as the primary driving factor (Figure 4C), with polybrene and centrifugation speed making smaller contributions to transduction efficiency (Table S2). Unexpectedly, within this design space, the model estimated no impact of the donor on the predicted transduction efficiency, (Figure 4D). This potentially highlights the robustness of the transduction conditions explored herein across different donors. The DoE model showed that the CD4:CD8 ratio within the CAR-positive fraction was primarily influenced by donor variability and LentiBOOST but not polybrene (Figure 4E). Furthermore, the addition of LentiBOOST further skewed the CAR-positive population toward a CD4 phenotype (Figure 4F). The remaining dependent variable, doubling time, was influenced only by donor type, with no significant contribution from centrifugation speed, LentiBOOST, or polybrene in its predictive model (Table S2).

Finally, based on the multi-response optimization of the DoE model using the desirability function, the combination of process parameters that maximizes overall outcomes (CAR expression, viability, CD4:CD8 ratio, and doubling time) comprises a spinoculation process using LentiBOOST at a concentration of 1 $mg.mL^{-1}$, no addition of polybrene (0 $mg.mL^{-1}$), and a centrifugation speed of 2,000 g. From this point onward, this process was referred to as the “optimized process,” as opposed to the baseline process.

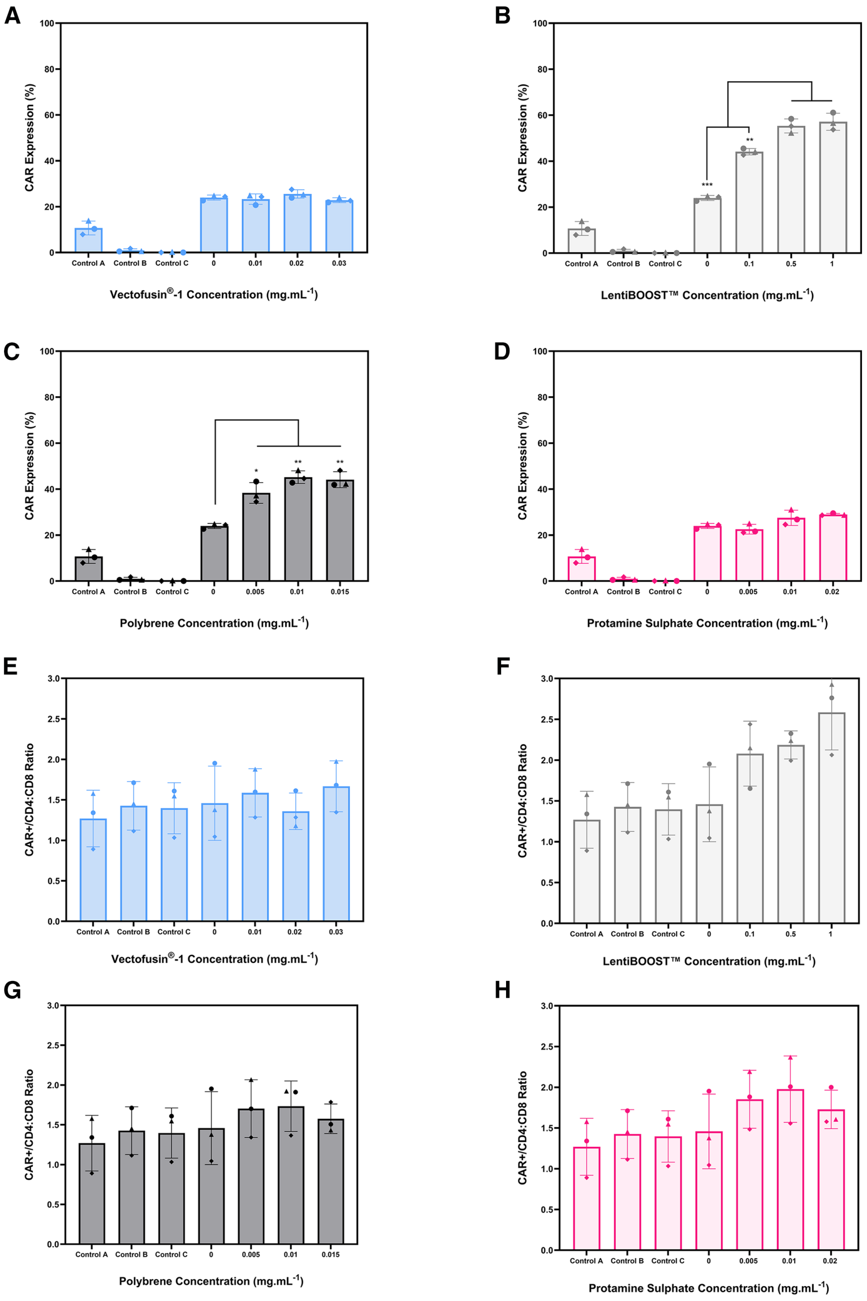

Figure 3. Impact of chemical factors on transduction

Representation of CAR transduction efficiency when (A) Vectofusin-1, (B) LentiBOOST, (C) polybrene, and (D) protamine sulfate were present during transduction at an MOI of 0.2. Impact of (E) Vectofusin-1, (F) LentiBOOST, (G) polybrene, and (H) protamine sulfate on CAR+/CD4:CD8 was also assessed. Bars represent the mean, and error bars indicate one standard deviation from three biological replicates (N = 3). Control A represents LV addition without spinoculation, control B represents spinoculation without LV, and control C lacked both LV and spinoculation. For controls B and C, the CD4:CD8 ratio obtained (marked with #) corresponds to non-transduced cells.

Impact of transduction processes on CAR T cell quality

To evaluate the potential impact of the different transduction processes at harvesting, an experiment featuring the entire upstream CAR T workflow was conducted (Figure S3). To assess the translational applicability of the optimized process, an additional group using the SXFM formulation under the same optimized transduction conditions was included.

As expected, the optimized process significantly enhanced transduction efficiency, nearly doubling CAR expression compared with the baseline (Figure 5A), from 21.4 ± 4.0% to 44.7 ± 7.4% at seeding (D0) and from 23.8 ± 4.7% to 46 ± 7.3% at harvest (D7). Notably, implementing the entire workflow in the SXFM formulation led to a further increase in CAR expression compared with the optimized process employing research-grade media (Roswell Park Memorial Institute [RPMI] supplemented with FBS). This effect was observed at both time points, D0 (44.7 ± 7.4% with RPMI *versus* 67.2 ± 4.4% with SXFM) and D7 (46 ± 7.3% *versus* 71.9 ± 2.4% for RPMI and SXFM, respectively). Additionally, across all experimental conditions, CAR expression levels remained stable between seeding and harvest.

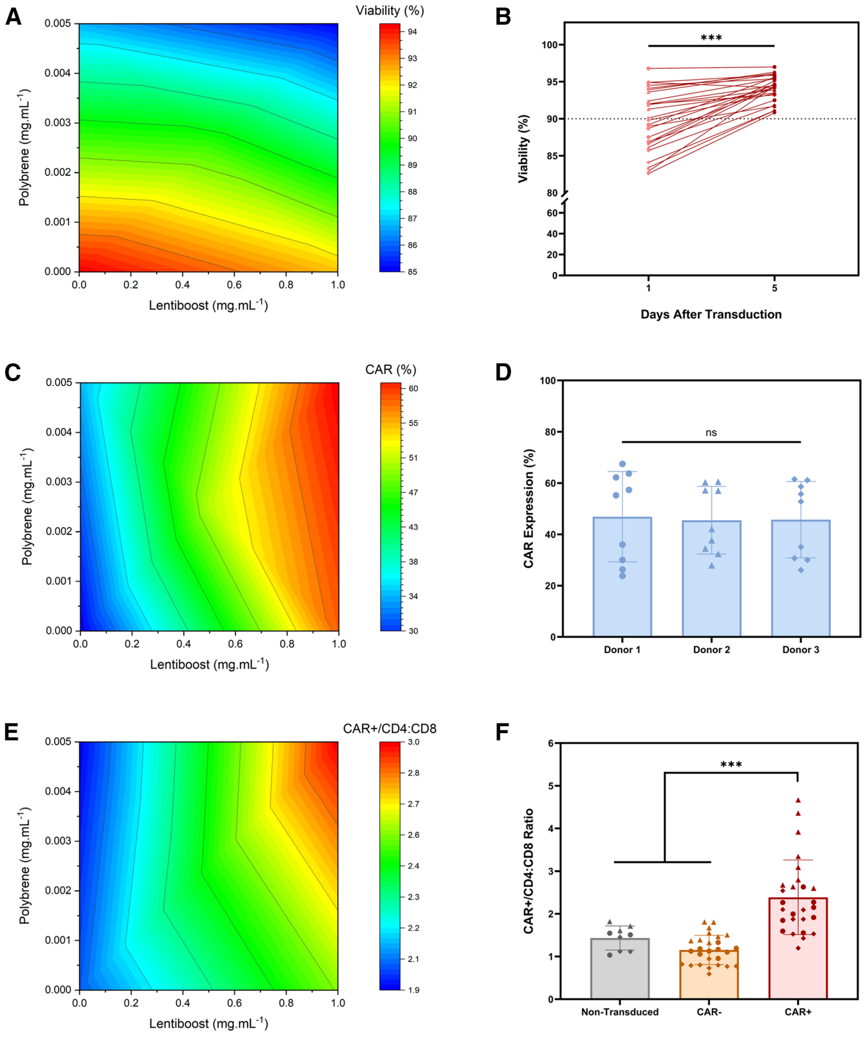

Figure 4. Evaluation of the optimal design space for transduction

Contour plot showing the impact of polybrene and LentiBOOST on (A) cell viability, with corresponding (B) viability percentages mapped across the design space throughout the experiment. Contribution of polybrene and LentiBOOST on (C) CAR expression (contour plot) and (D) across multiple donors. Representation of the effects of polybrene and LentiBOOST on (E) the CAR$^+$/CD4:CD8 ratio (contour plot), together with a comparison of CD4:CD8 ratios in non-transduced cells and CAR$^-$ and CAR$^+$ populations under the conditions defined by this design space. Data are presented as mean ± standard deviation in (B), (D), and (F), with individual DoE values shown as dots.

Analysis of the vector copy number (VCN) at day 7 showed that SXFM led to a significant increase in copy number (4.07 ± 0.21 copies.cell^{-1}) compared with the baseline process (1.35 ± 0.09 copies.cell^{-1}) and the optimized process (1.90 ± 0.32 copies.cell^{-1}), which were performed with RPMI supplemented with FBS (Figure 5B).

Neither the transduction process nor the switch to an SXFM formulation resulted in notable changes in the CAR+/CD4:CD8 ratio across experimental groups (Figure 5C). The observed variability within each group was largely driven by a single donor (indicated by the triangle), with this effect present at both seeding and harvest time points. This pattern was consistent across conditions, suggesting that the process modifications did not introduce additional variability in the CAR+/CD4:CD8 ratio. A decrease in the ratio was observed between D0 and D7 across the experimental groups, in line with the expected growth kinetics of CD4 and CD8 cells.[39,40]

No significant differences in doubling time across experimental groups were observed, indicating that neither process changes nor the shift to SXFM had an impact on overall cell growth kinetics (Figure 6A). In this study, doubling times ranged within a relatively narrow window of approximately 36–50 h.

Analysis of metabolite production and consumption rates revealed condition-dependent differences in nutrient uptake and by-product accumulation (Figure 6B). While glucose consumption remained comparable across conditions (ranging between 46.5 and 119.67 pmol.cell^{-1}.day^{-1}), lactate production was statistically higher in the optimized process groups (110.5 ± 29.4 and 209.4 ± 31.0 pmol.cell^{-1}.day^{-1} for the optimized and optimized SXFM groups, respectively), suggesting increased lactate production per mole of glucose consumed. Ammonia production rates remained comparable across all conditions (ranging from 9.26 to 12.48 pmol.cell^{-1}.day^{-1}), indicating similarities in the rates at which amino acids are catabolized. These findings suggest that, while SXFM supports growth kinetics comparable to those observed with FBS-based formulations in static culture, it may lead to a distinct metabolic profile in the expanded CAR T cells.

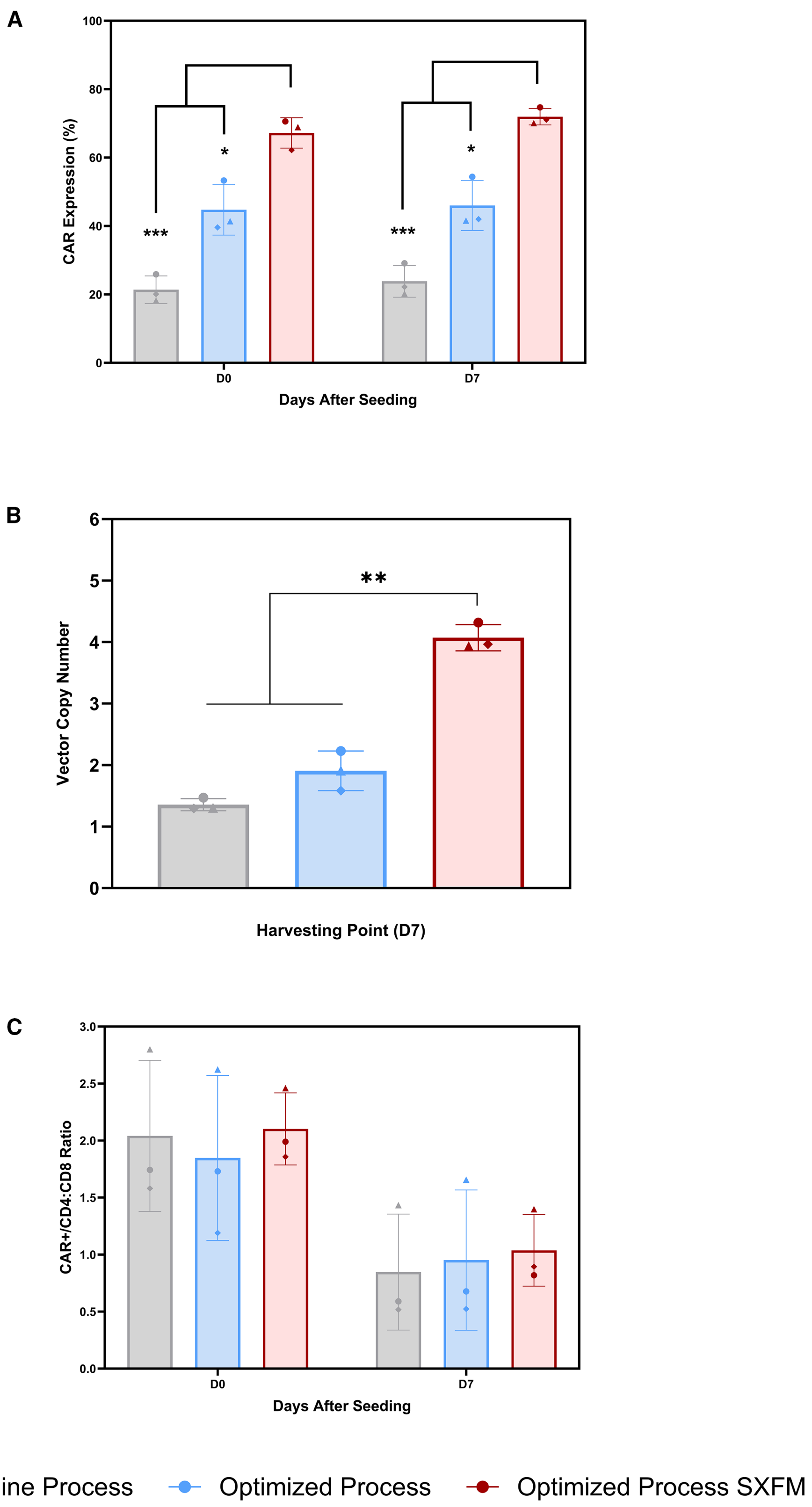

(legend on next page)

Functional evaluation via a cytotoxicity assay revealed that CAR T cells generated under both optimized process and optimized process SXFM conditions retained the ability to specifically lyse target NALM-6 cells, a human B cell precursor acute lymphoblastic leukemia expressing the CD19 receptor (Figure 6C). These findings indicate that functional activity is preserved despite changes in the transduction process and media formulation.

Regarding the immunophenotype at seeding and harvesting, it was observed that neither the transduction process nor the medium formulation used had an impact on the CD8 subsets (naive, central memory, effector memory, and terminally differentiated) (Figures 7A and 7B). At the time of harvest, the CD8 population was predominantly composed of central memory cells (~80%), followed by naive cells (~15%). In contrast, more differentiated and exhausted phenotypes, such as effector and terminally differentiated cells, remained below 5%. Activation and exhaustion marker expression was comparable across experimental conditions at both time points assessed (Figure 7C).

To investigate the transcriptomic impact of the transduction process, RNA sequencing (RNA-seq) was performed on the CAR T cells generated across the different experimental conditions: baseline, optimized, and optimized with SXFM (Figures 8A–8D). The heatmap of the 30 genes with the highest variance in expression revealed similarity between samples generated under the baseline and optimized processes across all three donors (Figure 8A). In contrast, samples from the optimized process using SXFM displayed a distinct gene expression profile. This difference was further supported by principal component analysis (PCA) (Figure 8B), which showed clear separation of the SXFM group along PC1 (the principal component capturing the largest source of variance). These findings strongly suggest that PC1 captures the influence of medium formulation on the transcriptome. At the same time, PC2 likely reflects donor-specific variability, as samples from the same donor clustered along the *y* axis regardless of the experimental condition used.

To assess transcriptional differences introduced by the optimized process compared with the baseline process, a differential gene expression analysis was performed (Figure 8C). This analysis identified only five genes (*RNA5S1*, *THY1*, *CHRNA6*, *CCR8*, and *RIPOR1*) that were significantly upregulated, with no genes found to be downregulated between the two groups.

To further test the hypothesis that PC1 captures transcriptomic variation primarily driven by medium formulation, a Gene Ontology (GO) term enrichment analysis was conducted to identify biological processes associated with genes upregulated or downregulated along this principal component (Figure 8D). The analysis revealed significant enrichment of biosynthetic and metabolic pathways, indicating that differences in medium composition likely account for the metabolic gene expression signatures observed along PC1. These findings support the interpretation that PC1 is predominantly influenced by the culture medium's impact on cellular metabolism.

DISCUSSION

With the rapid growth of clinical trials in the cell and gene therapy field, particularly those involving *ex vivo* gene modification, the gene transfer step has emerged as one of the major manufacturing bottlenecks.[41–46] This challenge results not only from the technical complexity of vector production and delivery but also from the substantial costs associated with these steps. These include batch-to-batch variability, low yields, and the high costs associated with plasmid DNA and transfection reagents.[6,17] Optimizing the gene transfer step reduces vector use, thereby lowering manufacturing costs and helping to broaden patient access.

Given the wide range of infectious titer quantification methods reported in the literature, this study aimed to assess how cell type and retronectin-coating processes influence titer measurements. Given the disparity in infectious titer measurements between cell types (150–300×), this study demonstrated the importance of matching, where possible, the titration conditions with those used during the transduction assay. Furthermore, it highlighted the critical need to disclose titration conditions in a manner that contextualizes the calculated MOI. In this study, we adopted an MOI of 0.2 and retronectin-coated plates, using the same prequalified donors used during the titer determination step.

Attempts to increase transduction efficiency for CAR T and hematopoietic stem cell (HSC) manufacturing have relied mostly on two approaches: physical and chemical methods.[19–21,23,24,26,27,30,32,35,47–54] Within the physical methods, spinoculation has been shown to enhance transduction through several mechanisms.[21,27,47,48] First, centrifugation increases contact between cells and viral particles by reducing the diffusion barrier of the vector in liquid, which is considered one of the main rate-limiting steps for particle adsorption to the cell surface.[21,23] In addition to these physical effects, spinoculation has been reported to trigger cytoskeletal rearrangements that facilitate receptor mobilization, viral entry, and subsequent post-entry steps.[26] Concerning potential toxicity, published studies have reported conflicting findings, with some showing no detrimental effects and others suggesting a negative impact on cell health.[30,48]

This study showed that varying the cell concentration ($2–4 \times 10^6$ cells.mL^{-1}) and spinoculation volume (2–4 mL) had no significant effect on transduction efficiency. However, both the speed and duration of the spinoculation cycle were demonstrated to impact transduction. Centrifugation speeds above 2000 g were shown to enhance

Figure 5. Assessing CAR-T cell quality under varying transduction conditions
(A) CAR expression, (B) vector copy number at harvest, and (C) CD4:CD8 ratios of CAR+ populations when manufactured across the different experimental conditions (baseline, optimized, and optimized process in SXFM). Bars represent the mean ± standard deviation obtained from three biological replicates (*N* = 3).

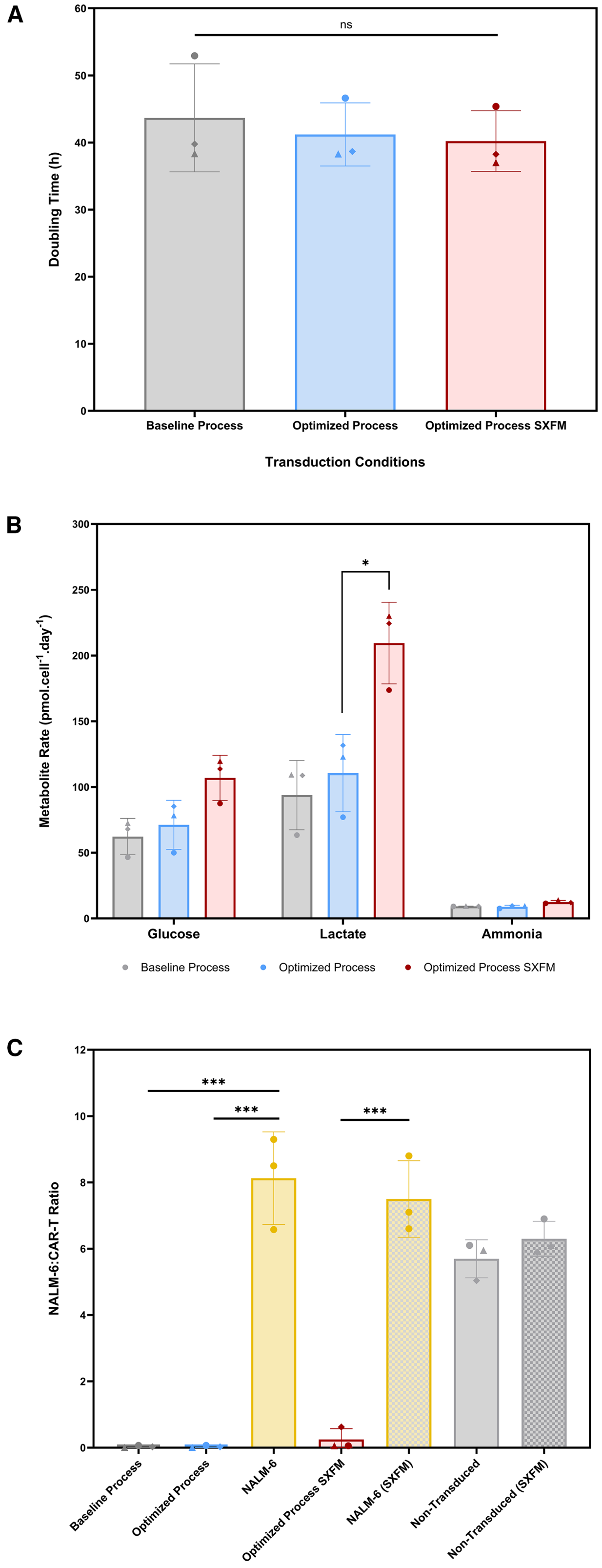

Figure 6. Evaluating the impact of transduction approaches on growth kinetics, metabolism and cytotoxic profile

(A) Doubling time, (B) metabolic production/consumption rates, and (C) *in vitro* cytotoxicity analysis across the different experimental conditions (baseline, optimized, and optimized process in SXFM). Bars represent the mean, and error bars represent one standard deviation, with individual replicates indicated by different symbols ($N = 3$).

transduction compared with lower speeds, and extending the spin duration to 80 min also led to significant improvements in transduction efficiency. These findings are consistent with previous reports and may be explained by the enhanced sedimentation of viral particles, which are known to remain largely suspended even when spinoculation is performed at forces close to 1000 g.[23,48]

Chemical methods, most commonly using polycations or poloxamers, have also been extensively studied due to their ability to reduce the electrostatic repulsion between negatively charged cell membranes and viral particles or to enhance membrane fluidity, thereby enabling viral vector diffusion.[30,31,47,55] Within this category, retronectin can be considered a separate class, as it does not rely on charge neutralization or modulation of membrane fluidity, unlike polycations or poloxamers. Instead, it enhances transduction by co-localizing viral particles and target cells, binding the vector through its heparin-binding domain and engaging integrins such as VLA-4 and VLA-5 on the cell surface to promote efficient vector-cell contact.[20,47] This work demonstrated that the use of retronectin can achieve a 1- to 3-fold increase in transduction efficiency compared with a control condition lacking this chemical enhancer and that the extent of this difference varies depending on the cell type used.

For polycations and poloxamers, the existing literature is limited, with most studies evaluating these molecules at a single concentration, thereby limiting the information available about their effects.[21,23,25,27,31,32,35,50,53,54] As this work demonstrates, LentiBOOST, a poloxamer, improves transduction efficiency in a dose-dependent manner. These findings are in line with previous research demonstrating that this dose-response relationship is molecule-dependent and likely impacted by cell type and overall transduction conditions.[20,30] This study identified LentiBOOST and polybrene, at concentrations of 0.1–1 $mg.mL^{-1}$ and 0.005–0.015 $mg.mL^{-1}$, respectively, as capable of increasing transduction efficiency by approximately 3-fold compared with conditions without these additives.

Given the potential impact of the physical and chemical conditions studied here on cell growth kinetics, doubling times were compared with those of the control groups. This analysis showed that, except for polybrene at concentrations above 0.005 $mg.mL^{-1}$, none of the tested conditions adversely affected growth kinetics, which is in agreement with several other studies.[20,30,53]

Because the one-factor-at-a-time experiments identified several parameters that enhanced transduction, the second phase of this study used a DoE approach to explore how these factors interact. This

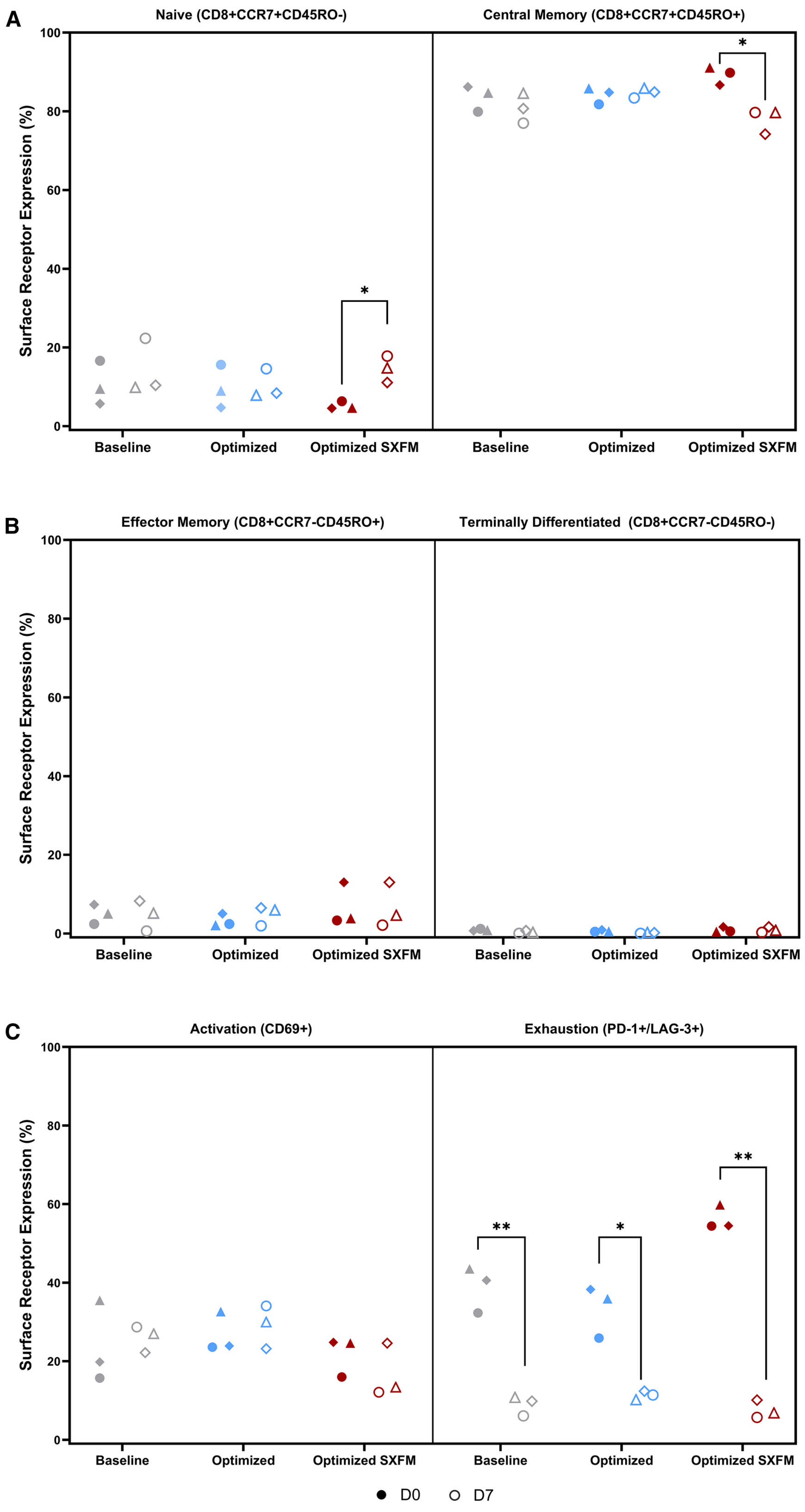

Figure 7. Effect of transduction methods on immunophenotype

Immunophenotypic characterization of (A) naive and central memory $CD8^+$ subsets, (B) effector and terminal effector $CD8^+$ subsets, and (C) activation and exhaustion markers across the different experimental conditions: baseline, optimized, and optimized process in SXFM. Biological replicates are plotted as individual values. (N = 3).

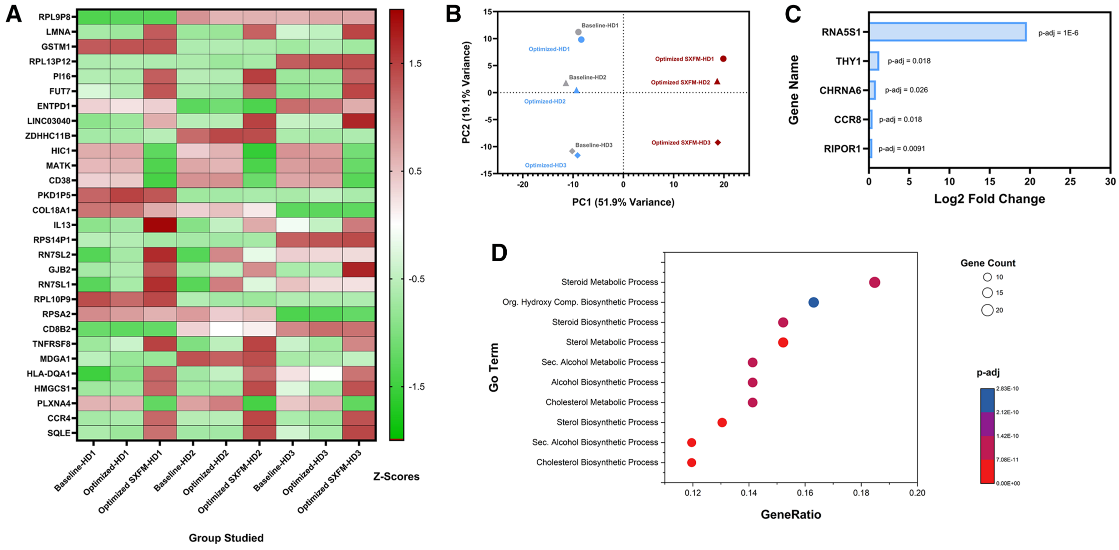

Figure 8. RNA-seq analysis comparing baseline, optimized, and optimized SXFM conditions

(A) Heatmap of the 30 most variable genes across all samples, (B) principal component analysis (PCA) plot showing sample clustering by condition, (C) genes with significantly different expression between baseline and optimized groups, (D) Gene Ontology (GO) enrichment analysis of genes contributing to the first principal component (PC1).

included testing different spinoculation speeds together with varying concentrations of polybrene and LentiBOOST to determine whether their effects were synergistic or antagonistic. Transduction efficiency was driven predominantly by LentiBOOST, with centrifugation speed providing a smaller incremental benefit. The DoE model revealed a clear difference between the two molecules, showing that polybrene reduces cell viability even at concentrations below 0.05 $mg.mL^{-1}$, whereas LentiBOOST exhibited minimal cytotoxic effect. As a result of this study, the DoE design space revealed a bias in the CD4:CD8 ratio of CAR-positive cells compared with the non-transduced population. Because this bias was confined to the CAR-positive fraction and was not observed in the CAR-negative cells that were equally exposed to the design space conditions, it strongly suggests that the shift arises from preferential transduction of $CD4^+$ cells rather than from direct cytotoxicity toward $CD8^+$ cells. In addition to these findings, this study demonstrated comparable transduction across different donors, indicating that the defined design space may be robust to biological variability. This represents a key strength of the work, as previous studies have reported substantial donor-to-donor and tissue-specific differences in transduction performance.[53,56,57] Given that this study employed T cells from healthy donors, further evaluation using patient-derived T cells would be necessary to assess the applicability of these findings in a clinical setting.

To validate the optimized processed identified in the DoE study, it was integrated into a CAR T cell manufacturing workflow, as described previously.[37,38,58–60] In parallel, to align the process with clinical manufacturing requirements, we assessed its performance by comparing three conditions: the baseline process, the optimized process established through the DoE, and the same optimized process conducted under SXFM conditions. This comparison showed that neither the transduction process nor the medium formulation had a detrimental effect on growth kinetics, which remained comparable across all experimental groups. Notably, the donor exhibiting the fastest growth kinetics remained the same across all conditions, reinforcing that donor-intrinsic factors, rather than experimental variables, were the primary source of variability in proliferation. The introduction of SXFM led to an increase in the lactate production rate compared with the optimized process ran with research-grade medium, which may indicate increased metabolic activity or a shift in glycolytic flux.[61] No statistically significant differences were observed between groups in terms of immunophenotype or cytotoxicity.

The transcriptomic analysis from the validation study showed only a small number of differentially expressed genes. The similarity in gene expression profiles observed in this study may be explained by two main factors. First, the T cell phenotype may be inherently robust to the process changes introduced during manufacturing. Second, the 9-day culture period under identical expansion conditions may lead to convergence of transcriptional states, masking earlier differences. It is therefore possible that transcriptomic analysis performed closer to the transduction time point would reveal greater differences. Bulk RNA-seq analysis of the final product showed no systematic transcriptome-level changes across the optimized transduction and expansion conditions tested. This indicates either a transient transcriptional response to transduction or that any early shifts in the transcriptomic profile are diluted by prolonged expansion, consistent with reports showing that even modest process stimuli can induce widespread transcriptional changes.[62]

The different transduction methods resulted in varying transduction efficiencies. The optimized process increased transduction

approximately 2-fold, while the same process conducted in SXFM achieved nearly a 3-fold improvement compared with the original baseline process. To further enhance transduction, an MOI escalation study was also performed (Figure S4). This study demonstrated that vector saturation was reached, as increasing the amount of vector beyond a certain point did not result in higher transduction efficiencies. These data suggest that heterogeneous primary populations contain cells that are relatively LV-resistant or non-permissive; consequently, adding more vector may primarily increase LV doses to permissive cells rather than enlarge the pool of transduced cells. In summary, this study identified key physical and chemical factors that influence viral transduction in CAR T manufacturing. Spinoculation speed and duration, together with the use of LentiBOOST or retronectin, significantly improved transduction, while polybrene was effective but limited by cytotoxicity. The optimized process approximately doubled transduction efficiency, with nearly a 3-fold improvement when applied under serum/xeno-free conditions, without affecting growth, immunophenotype, or cytotoxicity. These results indicate that enhancing cell-vector contact, rather than simply increasing vector dose, is key to improving transduction, reducing vector requirements and associated costs, and supporting the incorporation of these parameters into studies focused on increasing transduction efficiency. This study further demonstrates that gene transfer within the explored design space can be enhanced through both physical and chemical contributions. Accordingly, approaches that increase the frequency of vector-cell encounters or restrict both components within a confined volume are expected to further improve transduction efficiency. Such effects may be achieved, for example, in agitated systems that promote vector-cell interactions through enhanced mixing or by strategies that minimize the physical distance between vectors and cells, such as encapsulation- or confinement-based techniques.[63,64]

This study employs a QbD framework for optimizing LV-based transduction, showing that accurate titer determination using primary cells and a systematic understanding of physical and chemical factors during transduction are critical to improving performance. By identifying LentiBOOST as the main driver of efficiency in this experimental setup, centrifugation parameters as incremental enhancers, and polybrene as limited by toxicity, this study establishes an optimized process for LV-based transduction. This approach resulted in a 2-fold improvement in efficiency and nearly a 3-fold improvement when applied in SXFM, without compromising phenotype, function, or transcriptome. This QbD approach offers a transferable framework for optimizing gene delivery, enabling reduced vector usage and lowering the costs associated with transduction. To determine whether the observations reported here extend to alternative vector preparations, such as vectors carrying different transgenes, employing distinct pseudotypes, or produced using different purification strategies, the same systematic approach would need to be employed. Accordingly, the conclusions of this study are specific to the design space utilized herein. Direct comparisons with studies using alternative retroviral systems or differently prepared viral vectors should therefore be made with caution, as changes in vector design or production can introduce confounding effects that alter transduction behavior independently of the process parameters examined.

Although the findings reported herein were generated using a single clinically relevant serum-free medium formulation, it is important to acknowledge that alternative SXFMs are available and may yield CAR T cell products with distinct characteristics. Differences in nutrient composition, cytokine supplementation, and buffering capacity across SXFMs can influence T cell activation state, differentiation, and metabolism, which may affect transduction efficiency, expansion kinetics, immunophenotype, and cytolytic function. As such, the quantitative outcomes reported in this study should be interpreted within the context of the medium formulation employed. Importantly, however, the systematic framework and analytical approach described here are readily transferable and can be applied to evaluate and optimize transduction performance across alternative clinically relevant medium formulations.

MATERIALS AND METHODS

T cell isolation

T cells from three independent donors were isolated from leukopaks (BioIVT, Burgess Hill, UK) using Pan T cell isolation kits (Miltenyi Biotec, Cologne, Germany), following the manufacturer's protocol. Upon arrival, the contents of a leukopak were diluted 1:1 with MACS buffer, composed of 5% (v/v) MACS BSA stock solution and 95% (v/v) autoMACS rinsing solution (Miltenyi Biotec, Cologne, Germany). Peripheral blood mononuclear cells (PBMCs) were pelleted by centrifugation (400 g, 5 min) and resuspended in the same buffer at a concentration of 2.5×10^8 cells.mL^{-1}. The suspension was incubated sequentially with a biotin-conjugated Pan T cell antibody cocktail and Pan T cell MicroBeads (Miltenyi Biotec, Cologne, Germany), each for 5 min at 4°C. The cell mixture was then loaded into pre-rinsed LS columns (Miltenyi Biotec, Cologne, Germany), and the negatively selected fraction, containing the enriched T cell population, was collected. Cells were subsequently centrifuged at 400 g for 5 min and resuspended in CS10 cryopreservation medium (BioLife Solutions, Bothell, WA, USA) at a final concentration of 50×10^6 cells.mL^{-1}. Samples were frozen overnight at −80°C using a CoolCell freezing container (Corning, Corning, NY, USA) before being transferred to liquid nitrogen storage within 24 h. Unless otherwise specified, all experiments in this study were performed using the same three donors throughout.

Medium formulations

T cells were expanded in two different media. The first consisted of RPMI medium (Thermo Fisher Scientific, Waltham, MA, USA) supplemented with 10% (v/v) FBS (Thermo Fisher Scientific, Waltham, MA, USA) and 2 mmol.L^{-1} L-glutamine (Thermo Fisher Scientific, Waltham, MA, USA). In the validation studies, CellGenix Advanced TCM (Sartorius, Göttingen, Germany), a SXFM formulation, was also evaluated. To support T cell activation and proliferation,

interleukin-2 (IL-2) at 30 IU.mL^{-1} (Miltenyi Biotec, Cologne, Germany) was added at each feeding step (Figure S3).

Thawing and activation

Cells were thawed using medium pre-warmed in a 37°C water bath. Following thawing, the cryoprotectant was removed by centrifugation at 400 g for 5 min, after which cells were resuspended to achieve a target density of 1–2 × 10^6 cells.mL^{-1} using one of the medium formulations described above. Activation was initiated 24 h post-thaw using TransAct (Miltenyi Biotec, Cologne, Germany) in the presence of IL-2 (Miltenyi Biotec, Cologne, Germany).

LV manufacturing

A LV preparation was used to perform CAR transgene knock-in. The vector was prepared starting with a 5-day expansion of HEK 293T cells (ATCC, Manassas, VA, USA) seeded at a density of 5,000 cells.cm^{-2}. For the transfection, the DNA plasmid ratio transfer vector:Gag-Pol:REV:VSVG was 4:2:1:1.2, with the transfer vector encoding a CAR gene, using a DNA:PEI ratio of 1:2.75. DNA (Aldevron, Fargo, ND, USA) and PEI (Sartorius, Göttingen, Germany) were mixed and incubated for 15 min at room temperature to allow for complexation. The solution was then added dropwise to the cells. A complete medium exchange was performed 6 h post-transfection. The supernatant was collected 48 h post-transfection, filtered through a 0.45 μM filter (Merck, Darmstadt, Germany), and concentrated using Lenti-X (TakaraBio, Shiga, Japan). Unless stated otherwise, infectious vector concentration was determined using an assay consisting of 2 × 10^6 cells.mL^{-1} in 6-well plates coated with 4 μg.cm^{-2} of retronectin (TakaraBio, Shiga, Japan).

Transduction

To investigate the spinoculation-based transduction process, a three-stage approach was employed: (1) a one-factor-at-a-time strategy to identify which physical and chemical parameters influence transduction efficiency, (2) a DoE approach to evaluate potential synergistic or antagonistic interactions between the parameters identified in stage one, and (3) confirmation of the optimized transduction conditions and assessment of their applicability to serum/xeno-free culture systems. Throughout this manuscript, the MOI used was 0.2, and the assay for infectivity determination relied on three primary T cell donors and 4 μg.cm^{-2} retronectin-coated plates. These donors were kept constant throughout this work, meaning the same set of healthy donors was used for vector infectivity determination and transduction studies.

Physical and chemical parameters screening

To identify which factors impact transduction, a baseline spinoculation process was used, featuring retronectin-coated 6-well plates (4 μg.cm^{-2}) and a centrifugation cycle (1000 g, 40 min, 33°C). The physical parameters evaluated in this study were cell concentration (2–4 × 10^6 cells.mL^{-1}), volume (2–4 mL) at which spinoculation was conducted, spinoculation speed (500–4000 g), and time (20–120 min). The chemical mediators tested were 10–30 μg.mL^{-1} of Vectofusin-1 (Miltenyi Biotec, Cologne, Germany), 0.1×–1.0 mg.mL^{-1} of LentiBOOST (Revvity, Waltham, MA, USA), 0.005–0.02 mg.mL^{-1} of polybrene (Sigma-Aldrich, Darmstadt, Germany), and 0.005–0.02 mg.mL^{-1} of protamine sulfate (Sigma-Aldrich, Darmstadt, Germany). Unless stated otherwise, the transduction step was performed using a cell suspension of 2 × 10^6 cells.mL^{-1} in a total volume of 2 mL per well. Following this step, the plates were incubated for 24 h and then seeded into suspension culture flasks at 0.5 × 10^6 cells.mL^{-1}, incubated at 37°C with 5% CO_2 for 6 days.

To evaluate the potential cytotoxic effects of some of the parameters, several controls were included in this study: control A, in which the LV preparation was added to the cells without spinoculation; control B, in which no LV was added but spinoculation was still performed; and control C, in which neither LV was added nor a spinoculation cycle was performed.

Evaluating interactions of physical and chemical parameters using a DoE approach

To assess the potential synergistic or antagonistic effects of the interactions between parameters identified as impact transduction, a DoE approach was conducted, featuring a design space outlined in Table S3. A full factorial design with three center points was chosen to evaluate the effects of three two-level factors (LentiBOOST, polybrene, and centrifugation speed) and one three-level factor (T cell donor). The baseline process was optimized using JMP's desirability function to maximize transduction efficiency. Center points were included at 0.5 mg.mL^{-1} LentiBOOST, 0.0025 mg.mL^{-1} polybrene, and 1500 RCF to assess model stability. Least squares linear models incorporating main effects and two-factor interactions were fitted for each response (CAR expression, viability at D0, and doubling time), with donor included as a fixed categorical factor. Model adequacy was assessed using R^2, RMSE, ANOVA p values, and residual diagnostics, confirming no evidence of lack of fit.

Similar to the initial screening stage, the transduction step was performed using a cell suspension of 2 × 10^6 cells.mL^{-1} in a total volume of 2 mL.well^{-1}. Once this step was completed, the plates were incubated for 24 h and then seeded in suspension culture flasks at 0.5 × 10^6 cells.mL^{-1}, incubated at 37°C and 5% CO_2 for 6 days.

Validation of the optimized spinoculation process

To assess the performance of the optimized process, a side-by-side comparison using different transduction processes was established, featuring the following groups: (1) baseline process (1000 g, 40 min, RPMI), (2) optimized process (2000 g, 80 min, 1 mg.mL^{-1} LentiBOOST, RPMI), and (3) optimized process in SXFM (2000 g, 80 min, 1 mg.mL^{-1} LentiBOOST, SXFM).

The growth kinetics, transduction efficiency, immunophenotype, cytotoxicity, and transcriptomic profiling across the three different processes were assessed.

Cell growth kinetics was evaluated in static conditions, as described previously.[37,38,58,65] Briefly, CAR T cells were seeded at 0.5 × 10^6 cells.mL^{-1} in T-75 flasks (Thermo Fisher Scientific,

Waltham, MA, USA), with a total working volume of 20 mL. The feeding strategy used herein consisted of a 50% medium addition, followed by a 25% medium top-up on days 3 and 4, respectively, and a 50% medium exchange on day 5 of the process (Figure S3). All medium formulations used in this work were as described above in the section "medium formulations." Each experimental group was conducted in triplicate, with each replicate using a different donor to account for biological variability.

Analytical techniques

Cell counts

Cell concentration and viability were determined using the NucleoCounter NC-3000 (Chemometec, Lillerød, Denmark) with NucleoView software, which applies image-based analysis. Measurements were performed using Via1-Cassettes (Chemometec, Lillerød, Denmark), pre-loaded with acridine orange (AO) and 4′,6-diamidino-2-phenylindole (DAPI). AO, a membrane-permeable dye, stains all cell nuclei, while DAPI selectively labels non-viable cells with compromised membranes. For each sampling point, 200 μL of cell suspension was transferred to a reaction tube and mixed using a vortex.

Metabolite analysis

Daily samples were collected during the expansion phase to assess concentrations of glucose, lactate, L-glutamine, and ammonia. To prepare the samples for analysis, cells and debris were removed by centrifugation at 350 g for 5 min, and the supernatants were stored at −80°C. Before analysis, frozen samples were thawed in a 37°C water bath and processed using the CuBiAn Bioanalyser (4BioCell GmbH, Bielefeld, Germany), following the manufacturer's instructions.

Immunophenotype

To characterize the T cell populations at both the start of the expansion and at harvest, flow cytometric analysis was conducted on freshly collected cell samples using a BD LSRFortessa X-20 flow cytometer (Becton, Dickinson and Company, Franklin Lakes, NJ, USA). The antibody panel included the following conjugated antibodies: CD3-BUV395, CD4-BUV805, CD8-APC-Cy7, CCR7-BV421, CD45RO-PE-Cy7 (all from BD Biosciences, Berkshire, UK); CD34-AlexaFluor647 (R&D Systems, Minneapolis, MN, USA), encoded within the anti-CD19 CAR construct; and CD69-FITC, PD-1-PE, LAG-3-BV711 (all from BioLegend, London, UK). A Live/Dead-UV511 stain (Invitrogen, Renfrewshire, UK) was used to exclude non-viable cells. This panel was designed to capture information on T cell subsets, differentiation status, activation, and exhaustion markers. For each condition, a minimum of 100, 000 events were acquired to ensure at least 10, 000 events within the CAR+ population. Fluorescence minus one (FMO) controls were included for CCR7, CD45RO, CAR, CD69, PD-1, and LAG-3 to support the gating strategy.

In vitro *cytotoxicity*

CAR+ T cells were isolated on the day of harvest using the CD34 magnetic isolation kit (Miltenyi Biotec, Cologne, Germany), following the manufacturer's instructions. Briefly, cell suspensions were incubated with FcR blocking reagent and CD34 MicroBeads (Miltenyi Biotec, Cologne, Germany), then washed and passed through a MACS LS column (Miltenyi Biotec, Cologne, Germany). Unbound cells were removed through washing steps, and the CD34+ fraction was eluted by removing the column from the magnetic field. The enriched CD34+ population was subsequently used for downstream CAR T cell assays.

An *in vitro* cytotoxicity assay was performed using the Incucyte S3 live-cell analysis system (Sartorius, Göttingen, Germany), following the manufacturer's protocol. Effector and target cells were co-cultured at a 1:1 ratio over 2 days, using CD19-positive NALM6 target cells (ATCC, Manassas, VA, USA) labeled with Incucyte Nuclight Green. Owing to their expression of CD19, NALM6 cells are widely used as a model for B-cell malignancies in anti-CD19 CAR T cell assays. Before use, NALM6 cells were thawed and expanded at a seeding density of 0.5×10^6 cells.mL^{-1}, with passaging carried out to maintain viable cell concentrations below 2.0×10^6 cells.mL^{-1}. Culture medium formulations were consistent with those used during CAR T cell manufacturing.

RNA-seq

Total RNA quantity and integrity were assessed using the Agilent 4200 TapeStation (Standard Total RNA assay), confirming RNA integrity number (RIN) values > 7.0 for all samples. mRNA libraries were prepared using the KAPA mRNA HyperPrep Kit (Roche, Basel, Switzerland), following the manufacturer's protocol. Poly-A mRNA was isolated using oligo(dT) magnetic beads, fragmented by chemical hydrolysis, and reverse-transcribed with in the presence of actinomycin D to ensure strand specificity. Second-strand synthesis incorporated dUTP, and A-tailing enabled adaptor ligation using xGen dual-index adaptors containing unique molecular identifiers (UMIs). Libraries were PCR-enriched for 11 cycles, validated for yield and absence of adapter dimers via Agilent TapeStation (HS DNA 1000 assay), and quantified using the Qubit dsDNA HS assay. Libraries were normalized to 4 nM and pooled for sequencing.

RNA-seq data were processed in R using Bioconductor packages, including DESeq2 and SARTools. Raw counts across 63,086 gene features were normalized using DESeq2's scaling method. A variance stabilizing transformation (VST) was applied prior to PCA. Differential expression analysis included model fitting, outlier detection using Cook's distance, dispersion estimation, and independent filtering. Log_2 fold changes and adjusted p values were computed for each comparison. Results were visualized using volcano plots, heatmaps, and GO enrichment analysis. Outputs were exported as CSV files and plotted in GraphPad Prism.

VCN

gDNA isolation

Genomic DNA (gDNA) was extracted from cells transduced with a LV encoding a second-generation CAR construct, as well as from

non-transduced control cells, using the QIAgen DNA mini kit (Qiagen, Hilden, Germany) according to the manufacturer's protocol. The concentration of extracted gDNA was measured using a NanoDrop One spectrophotometer (Thermo Fisher Scientific, Waltham, MA, USA).

ddPCR for VCN analysis

VCN was quantified by droplet digital PCR (ddPCR) using primers and a probe targeting the 4-1BB/CD3ζ junction of the CAR construct. The human *RPP30* gene served as a reference for copy number normalization, using previously published primer and probe sequences.[66] Primer specificity was cross-checked against the GRCh38.p14 human genome assembly using Primer-BLAST (NCBI). All oligonucleotides, including FAM- and HEX-labelled ZEN/Iowa Black FQ double-quenched probes, were synthesized by Integrated DNA Technologies (Coralville, Iowa, USA).

ddPCR was performed on the Bio-Rad QX200 system (Biorad, Hercules, CA, USA). Duplex reactions included 50 ng of gDNA, 1× ddPCR Supermix for Probes (no dUTP; Biorad, Hercules, CA, USA), 20 U HindIII-HF (NEB, R3104T), primers (900 nM each), and FAM- and HEX-labelled probes (250 nM each) in a final volume of 25 μL, adjusted with nuclease-free water (Thermo Fisher Scientific, Waltham, MA, USA). Reaction droplets were generated using Droplet Generation Oil for Probes (Biorad, Hercules, CA, USA) with the QX200 manual droplet generator, following the manufacturer's instructions. PCR amplification was performed on a Bio-Rad C1000 Touch thermocycler, with a ramp rate of 2°C/s and heated lid set at 104°C. The cycling protocol was as follows: 95°C for 10 min; 40 cycles of 94°C for 30 s and 59°C for 1 min; 98°C for 1 min; hold at 4°C. Droplets were analyzed using the QX200 droplet reader. CAR-positive events were detected via FAM fluorescence, and the *RPP30* reference signal via HEX. Fluorescence amplitude thresholds were manually set at 1,800 (FAM) and 1,600 (HEX). Bulk VCN values were calculated using QX Manager Standard Edition version 2.0.0 (Biorad, Hercules, CA, USA), with *RPP30* normalized to two copies per diploid genome. Final copy number values were adjusted based on the transduction efficiency for each experimental condition, as determined by flow cytometry.

Statistical analysis

Statistical analyses were performed using SPSS software (IBM, Armonk, NY). The underlying hypothesis of each experiment guided the choice of statistical tests. Where data failed to meet the assumptions required for parametric testing, appropriate non-parametric alternatives were employed. Repeated-measures ANOVA was used when normality assumptions were met, and the one-way Friedman test served as a non-parametric alternative when they were not. Post hoc comparisons were carried out using paired comparisons for parametric analyses and pairwise Wilcoxon signed-rank tests for non-parametric analyses, with Bonferroni correction applied in both cases. DoE analyses were conducted using JMP software (SAS Institute, Cary, NC). Statistical significance was defined as $p < 0.05$, with significance levels indicated as follows: $^{*}p < 0.05$, $^{**}p < 0.01$, $^{***}p < 0.001$, and $^{****}p < 0.0001$. Unless otherwise stated, values in the manuscript are presented as mean ± standard deviation.

Equations

Doubling time

$$t_d = \frac{Ln_2}{\mu} \qquad \text{Equation 1}$$

The numerator represents the natural log of 2, and μ denotes the specific growth rate (d^{-1}).

Growth rate

$$\mu = \frac{Ln\left(\frac{cx(t)}{cx(0)}\right)}{\Delta t} \qquad \text{Equation 2}$$

Here, μ denotes the specific growth rate (d^{-1}), while Cx(t) and Cx(0) correspond to the total cell count at the conclusion and initiation of the exponential growth phase, respectively. The variable t (d) represents time.

Fold increase

$$FI = \frac{cx(f)}{cx(0)} \qquad \text{Equation 3}$$

Cx(t) and Cx(0) denote the total cell count at the end and start of the process, respectively.

Metabolic production/consumption rate

$$q_{met} = \frac{\mu}{cx(0)} \times \frac{C_{met(t)} - C_{met(0)}}{e^{\mu t} - 1} \qquad \text{Equation 4}$$

In this context, *qmet* (pmol.cell^{-1}.d^{-1}) refers to the specific metabolic rate, while μ denotes the specific growth rate (d^{-1}). *Cmet (t)* and *Cmet (0)* indicate the metabolite concentrations (mmol.L^{-1}) at the end and start of the exponential growth phase, respectively. *Cx(0)* represents the total cell count at the beginning of the exponential phase, and *t* corresponds to time (d).

DATA AND CODE AVAILABILITY

The data supporting this study's findings are available from the corresponding author upon reasonable request.

ACKNOWLEDGMENTS

The authors acknowledge the funding and support of the European Union through the AIDPATH project (Funding Code: 101016909). This project also includes financial and in-kind support from a consortium of industrial users and sector organizations. Additional support was provided by a UKRI EPSRC Fellowship grant awarded to Q.A.R. (EP/V058266/1). The authors also acknowledge funding from the University College London – Cytiva Center of Excellence and the Engineering and Physical Sciences Research Council Prosperity Partnership "Smart Biomanufacturing for Genomic Medicines" grant (EP/X025446/1). The Wellcome Trust Translational Partnership Award via UCL's Therapeutic Innovation Networks Pilot Scheme 3 (214046/Z/18/Z) also supported this work. Further funding was provided by UCL's Institute of Healthcare Engineering Discovery Award in 2025 and the EPSRC-funded FAST CAR-T: Faster, Adaptive and Scalable Technologies For CAR-T Manufacture (EP/Z532770/1).

AUTHOR CONTRIBUTIONS

Conceptualization, P.S.C., D.J.S., Y.T., Q.A.R.; Formal analysis, P.S.C.; funding acquisition, P.S.C. and Q.A.R.; investigation, P.S.C., D.J.S., B.C.S., P.S., and S.M.; methodology, P.S.C., D.J.S, B.C.S., P.S., S.M., and Y.T.; supervision, Q.A.R.; visualization, P.S.C.; writing – original draft, P.S.C.; writing – review & editing, P.S.C., D.J.S., and Q.A.R. All authors have read and agreed to the published version of the manuscript.

DECLARATION OF INTERESTS

U.S. and M.E. were Sartorius employees at the time this work was performed.

SUPPLEMENTAL INFORMATION

Supplemental information can be found online at https://doi.org/10.1016/j.omta.2026.201691.

REFERENCES

1. Maude, S.L., Laetsch, T.W., Buechner, J., Rives, S., Boyer, M., Bittencourt, H., Bader, P., Verneris, M.R., Stefanski, H.E., Myers, G.D., et al. (2018). Tisagenlecleucel in Children and Young Adults with B-Cell Lymphoblastic Leukemia. N. Engl. J. Med. *378*, 439–448. https://doi.org/10.1056/NEJMoa1709866.

2. Papadouli, I., Mueller-Berghaus, J., Beuneu, C., Ali, S., Hofner, B., Petavy, F., Tzogani, K., Miermont, A., Norga, K., Kholmanskikh, O., et al. (2020). EMA Review of Axicabtagene Ciloleucel (Yescarta) for the Treatment of Diffuse Large B-Cell Lymphoma. Oncologist *25*, 894–902. https://doi.org/10.1634/theoncologist.2019-0646.

3. Wang, Y., Jain, P., Locke, F.L., Maurer, M.J., Frank, M.J., Munoz, J.L., Dahiya, S., Beitinjaneh, A.M., Jacobs, M.T., Mcguirk, J.P., et al. (2023). Brexucabtagene Autoleucel for Relapsed or Refractory Mantle Cell Lymphoma in Standard-of-Care Practice: Results From the US Lymphoma CAR T Consortium. J. Clin. Oncol. *41*, 2594–2606. https://doi.org/10.1200/JCO.22.01797.

4. Aschenbrenner, D.S. (2022). New Approval for Drug Treating Large B-Cell Lymphoma. Am. J. Nurs. *122*, 20–21. https://doi.org/10.1097/01.NAJ.0000890212.43727.ab.

5. Comisel, R.-M., Kara, B., Fiesser, F.H., and Farid, S.S. (2021). Gene therapy process change evaluation framework: Transient transfection and stable producer cell line comparison. Biochem. Eng. J. *176*, 108202. https://doi.org/10.1016/j.bej.2021.108202.

6. Comisel, R.-M., Kara, B., Fiesser, F.H., and Farid, S.S. (2021). Lentiviral vector bioprocess economics for cell and gene therapy commercialization. Biochem. Eng. J. *167*, 107868. https://doi.org/10.1016/j.bej.2020.107868.

7. Stibbs, D.J., Silva Couto, P., Takeuchi, Y., Rafiq, Q.A., Jackson, N.B., and Rayat, A.C.M.E. (2024). Quasi-perfusion studies for intensified lentiviral vector production using a continuous stable producer cell line. Mol. Ther. Methods Clin. Dev. *32*, 101264. https://doi.org/10.1016/j.omtm.2024.101264.

8. Stibbs, D.J., Silva Couto, P., Takeuchi, Y., Rafiq, Q.A., Jackson, N.B., and Rayat, A.C.M.E. (2024). Continuous manufacturing of lentiviral vectors using a stable producer cell line in a fixed-bed bioreactor. Mol. Ther. Methods Clin. Dev. *32*, 101209. https://doi.org/10.1016/j.omtm.2024.101209.

9. Couto, P.S., Stibbs, D.J., Rotondi, M.C., Takeuchi, Y., and Rafiq, Q.A. (2023). Scalable manufacturing of gene-modified human mesenchymal stromal cells with microcarriers in spinner flasks. Appl. Microbiol. Biotechnol. *107*, 5669–5685. https://doi.org/10.1007/s00253-023-12634-w.

10. Trainor, N., Purpura, K.A., Middleton, K., Fargo, K., Hails, L., Vicentini-Hogan, M., McRobie, C., Daniels, R., Densham, P., Gardin, P., et al. (2023). Automated production of gene-modified chimeric antigen receptor T cells using the Cocoon Platform. Cytotherapy *25*, 1349–1360. https://doi.org/10.1016/j.jcyt.2023.07.012.

11. Palani, H.K., Arunachalam, A.K., Yasar, M., Venkatraman, A., Kulkarni, U., Lionel, S.A., Selvarajan, S., Korula, A., Abraham, A., George, B., et al. (2023). Decentralized manufacturing of anti CD19 CAR-T cells using CliniMACS Prodigy®: real-world experience and cost analysis in India. Bone Marrow Transplant. *58*, 160–167. https://doi.org/10.1038/s41409-022-01866-5.

12. Zhu, F., Shah, N.N., Xu, H., Schneider, D., Orentas, R., Dropulic, B., Hari, P., and Keever-Taylor, C.A. (2016). CAR-T Cell Production Using the Clinimacs® Prodigy System. Blood *128*, 5724. https://doi.org/10.1182/blood.V128.22.5724.5724.

13. Geng, G., Xu, Y., Hu, Z., Wang, H., Chen, X., Yuan, W., and Shu, Y. (2025). Viral and non-viral vectors in gene therapy: current state and clinical perspectives. EBioMedicine *118*, 105834. https://doi.org/10.1016/j.ebiom.2025.105834.

14. Chicaybam, L., Sodre, A.L., Curzio, B.A., and Bonamino, M.H. (2013). An Efficient Low Cost Method for Gene Transfer to T Lymphocytes. PLoS One *8*, e60298. https://doi.org/10.1371/journal.pone.0060298.

15. Chicaybam, L., Barcelos, C., Peixoto, B., Carneiro, M., Limia, C.G., Redondo, P., Lira, C., Paraguassú-Braga, F., Vasconcelos, Z.F.M.D., Barros, L., et al. (2016). An Efficient Electroporation Protocol for the Genetic Modification of Mammalian Cells. Front. Bioeng. Biotechnol. *4*, 99. https://doi.org/10.3389/fbioe.2016.00099.

16. Hudecek, M., and Ivics, Z. (2018). Non-viral therapeutic cell engineering with the Sleeping Beauty transposon system. Curr. Opin. Genet. Dev. *52*, 100–108. https://doi.org/10.1016/j.gde.2018.06.003.

17. Labbé, R.P., Vessillier, S., and Rafiq, Q.A. (2021). Lentiviral Vectors for T Cell Engineering: Clinical Applications, Bioprocessing and Future Perspectives. Viruses *13*, 1528. https://doi.org/10.3390/v13081528.

18. Milone, M.C., and O'Doherty, U. (2018). Clinical use of lentiviral vectors. Leukemia *32*, 1529–1541. https://doi.org/10.1038/s41375-018-0106-0.

19. Uchida, N., Nassehi, T., Drysdale, C.M., Gamer, J., Yapundich, M., Demirci, S., Haro-Mora, J.J., Leonard, A., Hsieh, M.M., and Tisdale, J.F. (2019). High-Efficiency Lentiviral Transduction of Human CD34+ Cells in High-Density Culture with Poloxamer and Prostaglandin E2. Mol. Ther. Methods Clin. Dev. *13*, 187–196. https://doi.org/10.1016/j.omtm.2019.01.005.

20. Piovan, C., Marin, V., Scavullo, C., Corna, S., Giuliani, E., Bossi, S., Galy, A., Fenard, D., Bordignon, C., Rizzardi, G.P., et al. (2017). Vectofusin-1 Promotes RD114-TR-Pseudotyped Lentiviral Vector Transduction of Human HSPCs and T Lymphocytes. Mol. Ther. Methods Clin. Dev. *5*, 22–30. https://doi.org/10.1016/j.omtm.2017.02.003.

21. Zielske, S.P., and Gerson, S.L. (2002). Lentiviral Transduction of P140K MGMT into Human CD34+ Hematopoietic Progenitors at Low Multiplicity of Infection Confers Significant Resistance to BG/BCNU and Allows Selection in Vitro. Mol. Ther. *5*, 381–387. https://doi.org/10.1006/mthe.2002.0571.

22. Su, S., Betters, D.M., Ramanathan, M., Keyvanfar, K., Smith, A., Feng, X., Furutani, E., Carlsten, M., Lundqvist, A., and Childs, R. (2011). Optimizing Lentiviral Transduction of Human Natural Killer Cells. Blood *118*, 4714. https://doi.org/10.1182/blood.V118.21.4714.4714.

23. Bahnson, A.B., Dunigan, J.T., Baysal, B.E., Mohney, T., Atchison, R.W., Nimgaonkar, M.T., Ball, E.D., and Barranger, J.A. (1995). Centrifugal enhancement of retroviral mediated gene transfer. J. Virol. Methods *54*, 131–143. https://doi.org/10.1016/0166-0934(95)00035-S.

24. Ghassemi, S., Durgin, J.S., Nunez-Cruz, S., Patel, J., Leferovich, J., Pinzone, M., Shen, F., Cummins, K.D., Plesa, G., Cantu, V.A., et al. (2022). Rapid manufacturing of non-activated potent CAR T cells. Nat. Biomed. Eng. *6*, 118–128. https://doi.org/10.1038/s41551-021-00842-6.

25. O'Doherty, U., Swiggard, W.J., and Malim, M.H. (2000). Human Immunodeficiency Virus Type 1 Spinoculation Enhances Infection through Virus Binding. J. Virol. *74*, 10074–10080. https://doi.org/10.1128/JVI.74.21.10074-10080.2000.

26. Guo, J., Wang, W., Yu, D., and Wu, Y. (2011). Spinoculation Triggers Dynamic Actin and Cofilin Activity That Facilitates HIV-1 Infection of Transformed and Resting CD4 T Cells. J. Virol. *85*, 9824–9833. https://doi.org/10.1128/JVI.05170-11.

27. Radek, C., Bernadin, O., Drechsel, K., Cordes, N., Pfeifer, R., Sträßer, P., Mormin, M., Gutierrez-Guerrero, A., Cosset, F.-L., Kaiser, A.D., et al. (2019). Vectofusin-1 Improves Transduction of Primary Human Cells with Diverse Retroviral and Lentiviral Pseudotypes, Enabling Robust, Automated Closed-System Manufacturing. Hum. Gene Ther. *30*, 1477–1493. https://doi.org/10.1089/hum.2019.157.

28. Malach, P., Kay, C., Tinworth, C., Patel, F., Joosse, B., Wade, J., Rosa do Carmo, M., Donovan, B., Brugman, M., Montiel-Equihua, C., et al. (2023). Identification of a small molecule for enhancing lentiviral transduction of T cells. Mol. Ther. Methods Clin. Dev. *31*, 101113. https://doi.org/10.1016/j.omtm.2023.101113.

29. Najafi, S., Rahimpour, A., Ahmadieh, H., Tehrani, M.M., Khalilzad, M.A., Suri, F., and Ranjbari, J. (2024). The significance of chemical transfection/transduction enhancers in promoting the viral vectors-assisted gene delivery approaches: A focus

on potentials for inherited retinal diseases. Electron. J. Biotechnol. *72*, 29–40. https://doi.org/10.1016/j.ejbt.2024.07.005.

30. Delville, M., Soheili, T., Bellier, F., Durand, A., Denis, A., Lagresle-Peyrou, C., Cavazzana, M., Andre-Schmutz, I., and Six, E. (2018). A Nontoxic Transduction Enhancer Enables Highly Efficient Lentiviral Transduction of Primary Murine T Cells and Hematopoietic Stem Cells. Mol. Ther. Methods Clin. Dev. *10*, 341–347. https://doi.org/10.1016/j.omtm.2018.08.002.

31. Anastasov, N., Höfig, I., Mall, S., Krackhardt, A.M., and Thirion, C. (2016). Optimized Lentiviral Transduction Protocols by Use of a Poloxamer Enhancer, Spinoculation, and scFv-Antibody Fusions to VSV-G. Methods Mol. Biol. *1448*, 49–61. https://doi.org/10.1007/978-1-4939-3753-0_4.

32. Munisvaradass, R., Kumar, S., Govindasamy, C., Alnumair, K.S., and Mok, P.L. (2017). Human CD3+ T-Cells with the Anti-ERBB2 Chimeric Antigen Receptor Exhibit Efficient Targeting and Induce Apoptosis in ERBB2 Overexpressing Breast Cancer Cells. Int. J. Mol. Sci. *18*, 1797. https://doi.org/10.3390/ijms18091797.

33. Denning, W., Das, S., Guo, S., Xu, J., Kappes, J.C., and Hel, Z. (2013). Optimization of the Transductional Efficiency of Lentiviral Vectors: Effect of Sera and Polycations. Mol. Biotechnol. *53*, 308–314. https://doi.org/10.1007/s12033-012-9528-5.

34. Abbasalipour, M., Khosravi, M.A., Zeinali, S., Khanahmad, H., Karimipoor, M., and Azadmanesh, K. (2019). Improvement of K562 Cell Line Transduction by FBS Mediated Attachment to the Cell Culture Plate. BioMed Res. Int. *2019*, 9540702. https://doi.org/10.1155/2019/9540702.

35. Cribbs, A.P., Kennedy, A., Gregory, B., and Brennan, F.M. (2013). Simplified production and concentration of lentiviral vectors to achieve high transduction in primary human T cells. BMC Biotechnol. *13*, 98. https://doi.org/10.1186/1472-6750-13-98.

36. Lipsitz, Y.Y., Timmins, N.E., and Zandstra, P.W. (2016). Quality cell therapy manufacturing by design. Nat. Biotechnol. *34*, 393–400. https://doi.org/10.1038/nbt.3525.

37. Hood, T., Slingsby, F., Sandner, V., Geis, W., Schmidberger, T., Bevan, N., Vicard, Q., Hengst, J., Springuel, P., Dianat, N., et al. (2024). A quality-by-design approach to improve process understanding and optimise the production and quality of CAR-T cells in automated stirred-tank bioreactors. Front. Immunol. *15*, 1335932. https://doi.org/10.3389/fimmu.2024.1335932.

38. Hood, T., Springuel, P., Slingsby, F., Sandner, V., Geis, W., Schmidberger, T., Bevan, N., Vicard, Q., Hengst, J., Dianat, N., et al. (2025). Establishing a scalable perfusion strategy for the manufacture of CAR -T cells in stirred-tank bioreactors using a quality-by-design approach. Bioeng. Transl. Med. *10*, e10753. https://doi.org/10.1002/btm2.10753.

39. Li, C.-R., Santoso, S., and Lo, D.D. (2007). Quantitative analysis of T cell homeostatic proliferation. Cell. Immunol. *250*, 40–54. https://doi.org/10.1016/j.cellimm.2008.01.003.

40. Kaech, S.M., Wherry, E.J., and Ahmed, R. (2002). Effector and memory T-cell differentiation: implications for vaccine development. Nat. Rev. Immunol. *2*, 251–262. https://doi.org/10.1038/nri778.

41. Couto, P.S., Shatirishvili, G., Bersenev, A., and Verter, F. (2019). First decade of clinical trials and published studies with mesenchymal stromal cells from umbilical cord tissue. Regen. Med. *14*, 309–319. https://doi.org/10.2217/rme-2018-0171.

42. Couto, P.S., Bersenev, A., and Verter, F. (2017). The first decade of advanced cell therapy clinical trials using perinatal cells (2005–2015). Regen. Med. *12*, 953–968. https://doi.org/10.2217/rme-2017-0066.

43. Verter, F., Couto, P.S., and Bersenev, A. (2018). A Dozen Years of Clinical Trials Performing Advanced Cell Therapy with Perinatal Cells. Future Sci. OA *4*. https://doi.org/10.4155/fsoa-2018-0085.

44. Rittié, L., Athanasopoulos, T., Calero-Garcia, M., Davies, M.L., Dow, D.J., Howe, S.J., Morrison, A., Ricciardelli, I., Saudemont, A., Jespers, L., et al. (2019). The Landscape of Early Clinical Gene Therapies outside of Oncology. Mol. Ther. *27*, 1706–1717. https://doi.org/10.1016/j.ymthe.2019.09.002.

45. Arabi, F., Mansouri, V., and Ahmadbeigi, N. (2022). Gene therapy clinical trials, where do we go? An overview. Biomed. Pharmacother. *153*, 113324. https://doi.org/10.1016/j.biopha.2022.113324.

46. Silva Couto, P., Stibbs, D.J., Sanchez, B.C., Khalife, R., Panagopoulou, T.I., Barnes, B., George, V., Taghizadeh, R.R., and Rafiq, Q.A. (2024). Generating suspension-adapted human mesenchymal stromal cells (S-hMSCs) for the scalable manufacture of extracellular vesicles. Cytotherapy *26*, 1532–1546. https://doi.org/10.1016/j.jcyt.2024.06.011.

47. Rajabzadeh, A., Hamidieh, A.A., and Rahbarizadeh, F. (2021). Spinoculation and retronectin highly enhance the gene transduction efficiency of Mucin-1-specific chimeric antigen receptor (CAR) in human primary T cells. BMC Mol. Cell Biol. *22*, 57. https://doi.org/10.1186/s12860-021-00397-z.

48. Kim, Y., Lee, D.Y., Choi, J.U., Park, J.S., Lee, S.M., Kang, C.H., and Park, C.H. (2023). Optimized conditions for gene transduction into primary immune cells using viral vectors. Sci. Rep. *13*, 12365. https://doi.org/10.1038/s41598-023-39597-2.

49. De Lageneste, M. (2020). Closed and automated CAR T cell lentiviral transduction by spinoculation. Cytotherapy *22*, S184. https://doi.org/10.1016/j.jcyt.2020.03.387.

50. Remley, V.A., Jin, J., Sarkar, S., Moses, L., Prochazkova, M., Cai, Y., Shao, L., Liu, H., Fuksenko, T., Jin, P., et al. (2021). High efficiency closed-system gene transfer using automated spinoculation. J. Transl. Med. *19*, 474. https://doi.org/10.1186/s12967-021-03126-4.

51. Yang, S., Rosenberg, S.A., and Morgan, R.A. (2008). Clinical-scale Lentiviral Vector Transduction of PBL for TCR Gene Therapy and Potential for Expression in Less-differentiated Cells. J. Immunother. *31*, 830–839. https://doi.org/10.1097/CJI.0b013e31818817c5.

52. O'Doherty, U., Swiggard, W.J., and Malim, M.H. (2000). Human immunodeficiency virus type 1 spinoculation enhances infection through virus binding. J. Virol. *74*, 10074–10080. https://doi.org/10.1128/jvi.74.21.10074-10080.2000.

53. Lo Presti, V., Cornel, A.M., Plantinga, M., Dünnebach, E., Kuball, J., Boelens, J.J., Nierkens, S., and van Til, N.P. (2021). Efficient lentiviral transduction method to gene modify cord blood CD8+ T cells for cancer therapy applications. Mol. Ther. Methods Clin. Dev. *21*, 357–368. https://doi.org/10.1016/j.omtm.2021.03.015.

54. Millington, M., Arndt, A., Boyd, M., Applegate, T., and Shen, S. (2009). Towards a Clinically Relevant Lentiviral Transduction Protocol for Primary Human CD34+ Hematopoietic Stem/Progenitor Cells. PLoS One *4*, e6461. https://doi.org/10.1371/journal.pone.0006461.

55. Silva Couto, P., Molina, S.A., O'Sullivan, D., O'Neill, L., Lyness, A.M., and Rafiq, Q.A. (2023). Understanding the impact of bioactive coating materials for human mesenchymal stromal cells and implications for manufacturing. Biotechnol. Lett. *45*, 1013–1027. https://doi.org/10.1007/s10529-023-03369-9.

56. Couto, P.S., Stibbs, D.J., Rotondi, M.C., Khalife, R., Wolf, D., Takeuchi, Y., and Rafiq, Q.A. (2024). Biological differences between adult and perinatal human mesenchymal stromal cells and their impact on the manufacturing processes. Cytotherapy *26*, 1429–1441. https://doi.org/10.1016/j.jcyt.2024.05.020.

57. Ling, C., Bhukhai, K., Yin, Z., Tan, M., Yoder, M.C., Leboulch, P., Payen, E., and Srivastava, A. (2016). High-Efficiency Transduction of Primary Human Hematopoietic Stem/Progenitor Cells by AAV6 Vectors: Strategies for Overcoming Donor-Variation and Implications in Genome Editing. Sci. Rep. *6*, 35495. https://doi.org/10.1038/srep35495.

58. Costariol, E., Rotondi, M.C., Amini, A., Hewitt, C.J., Nienow, A.W., Heathman, T.R.J., and Rafiq, Q.A. (2020). Demonstrating the manufacture of human car-t cells in an automated stirred-tank bioreactor. Biotechnol. J. *15*, 2000177. https://doi.org/10.1002/biot.202000177.

59. Springuel, P., Hood, T., Slingsby, F., Schmidberger, T., Bevan, N., Dianat, N., Hengst, J., and Rafiq, Q.A. (2025). Optimising and adapting perfusion feeds in serum-free medium to intensify CAR-T cell expansion in stirred-tank bioreactors. Front. Bioeng. Biotechnol. *13*, 1593895. https://doi.org/10.3389/fbioe.2025.1593895.

60. Silva Couto, P., Stibbs, D.J., Springuel, P., Schultz, U., Effenberger, M., Goldrick, S., Navarro-Velázquez, S., Juan, M., Herbst, L., Nießing, B., et al. (2025). Impact of serum/Xeno-free medium and cytokine supplementation on CAR-T cell therapy manufacturing in stirred tank bioreactors. Biotechnol. J. *20*, e70114. https://doi.org/10.1002/biot.70114.

61. Kozlov, A.M., Lone, A., Betts, D.H., and Cumming, R.C. (2020). Lactate preconditioning promotes a HIF-1α-mediated metabolic shift from OXPHOS to glycolysis in normal human diploid fibroblasts. Sci. Rep. *10*, 8388. https://doi.org/10.1038/s41598-020-65193-9.

62. Cano-Gamez, E., Soskic, B., Roumeliotis, T.I., So, E., Smyth, D.J., Baldrighi, M., Willé, D., Nakic, N., Esparza-Gordillo, J., Larminie, C.G.C., et al. (2020). Single-cell transcriptomics identifies an effectorness gradient shaping the response of CD4+ T cells to cytokines. Nat. Commun. *11*, 1801. https://doi.org/10.1038/s41467-020-15543-y.

63. Park, J.-O., Park, S.-H., and Hong, S.-T. (2009). A New Method for Transduction of Mesenchymal Stem Cells Using Mechanical Agitation. Mol. Cells *28*, 515–520. https://doi.org/10.1007/s10059-009-0146-4.

64. Moore, N., Chevillet, J.R., Healey, L.J., McBrine, C., Doty, D., Santos, J., Teece, B., Truslow, J., Mott, V., Hsi, P., et al. (2019). A Microfluidic Device to Enhance Viral Transduction Efficiency During Manufacture of Engineered Cellular Therapies. Sci. Rep. *9*, 15101. https://doi.org/10.1038/s41598-019-50981-9.

65. Costariol, E., Rotondi, M., Amini, A., Hewitt, C.J., Nienow, A.W., Heathman, T.R.J., Micheletti, M., and Rafiq, Q.A. (2019). Establishing the scalable manufacture of primary human T-cells in an automated stirred-tank bioreactor. Biotechnol. Bioeng. *116*, 2488–2502. https://doi.org/10.1002/bit.27088.

66. Wang, W., Al-Hajj, M., and Alavi, A.S. (2023). Detection and quantification of integrated vector copy number by multiplex droplet digital PCR in dual-transduced CAR T cells. Mol. Ther. Methods Clin. Dev. *30*, 403–410. https://doi.org/10.1016/j.omtm.2023.07.003.

OMTA, Volume *34*

Supplemental information

Impact of physical and chemical parameters on spinoculation for chimeric antigen receptor T cell manufacturing using a quality-by-design approach

Pedro Silva Couto, Dale J. Stibbs, Braulio Carrillo Sanchez, Pierre Springuel, Syd McLean, Ursula Schultz, Manuel Effenberger, Yasuhiro Takeuchi, and Qasim A. Rafiq

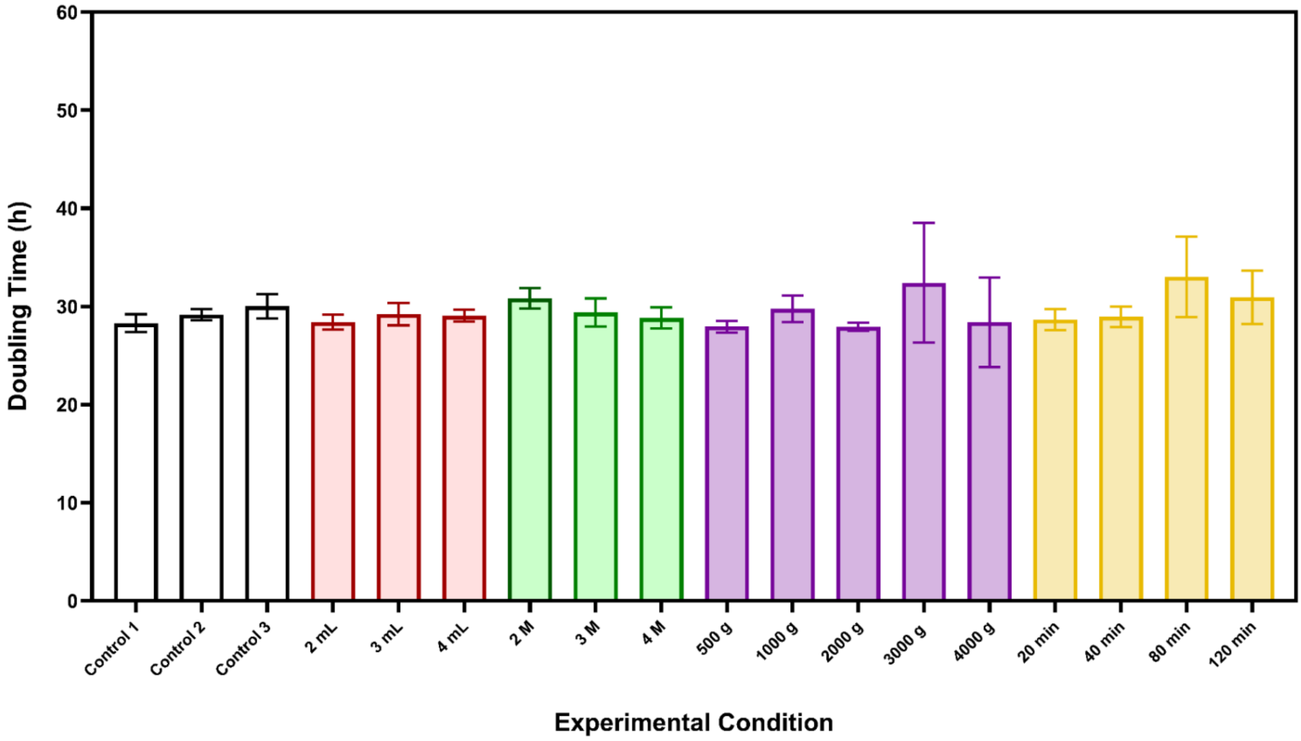

FIGURE S1-Representation of the doubling time across conditions tested in the evaluation of physical parameters on transduction efficiency via spinoculation (N=3). Bars representing mean and standard deviation represented as error bars.

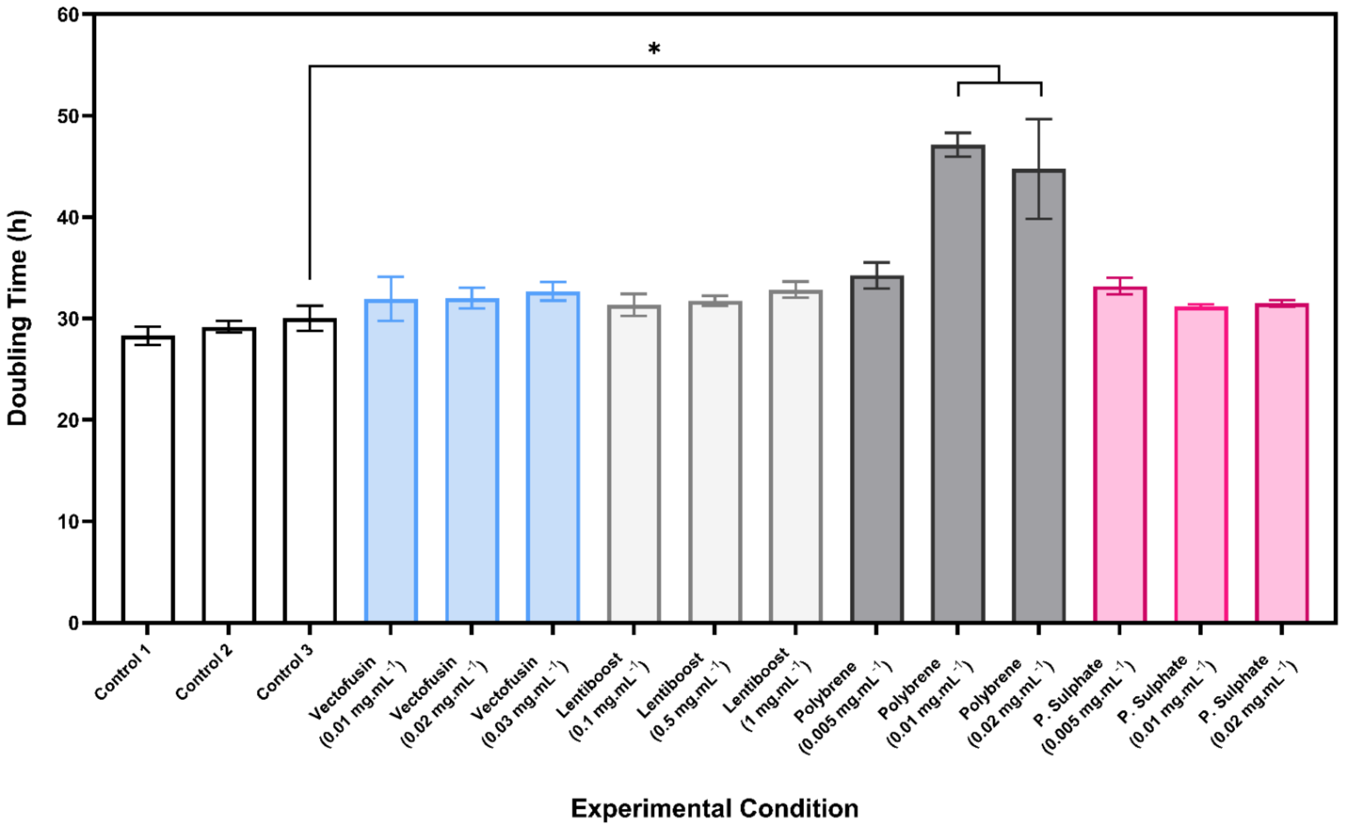

FIGURE S2-Representation of the doubling time across conditions tested in the evaluation of chemical parameters on transduction efficiency via spinoculation (N=3). Bars representing mean and standard deviation represented as error bars.

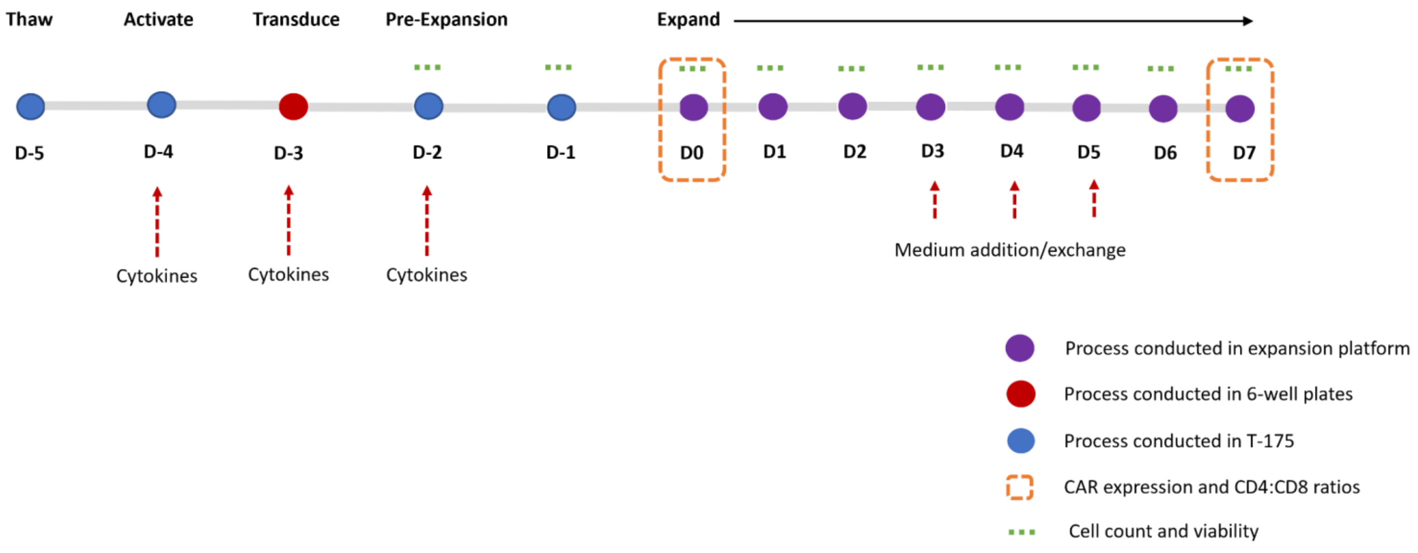

FIGURE S3-Manufacturing process used to compare the baseline process to the optimised process and optimised process when performed with serum/xeno-free medium.

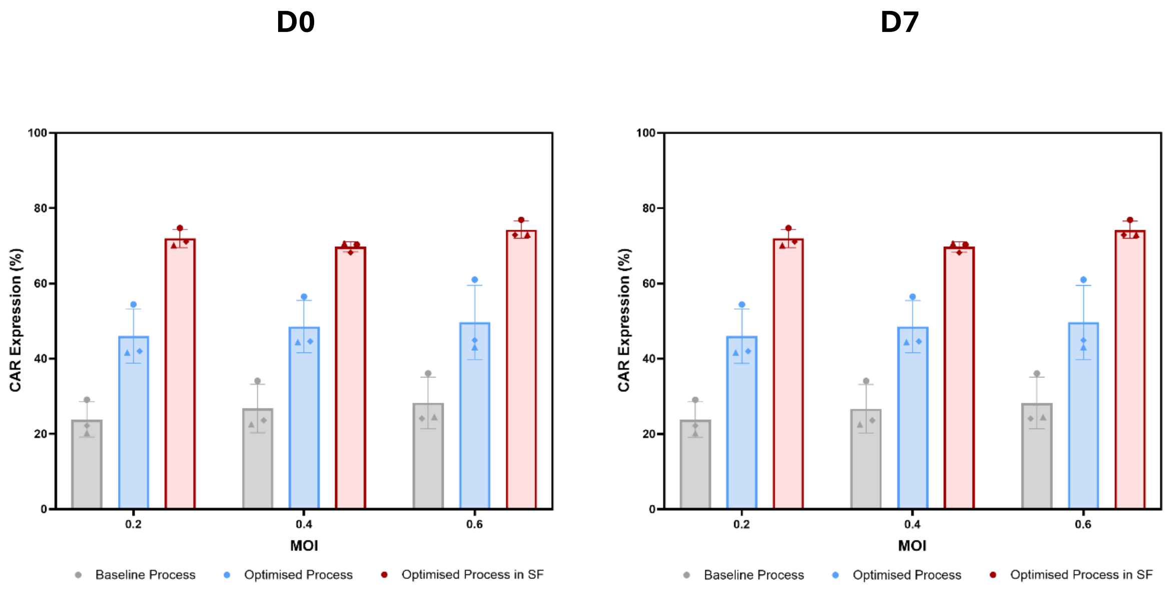

FIGURE S4-Evaluation of the impact of different MOIs across the different transduction processes (N=3). Bars representing mean and standard deviation represented as error bars.

TABLE S1-List of functional titre values obtained across biological donors and titration methods.

Titration Method	Donor	Replicate	Titre (TU.mL^{-1})
Jurkat	1	A	2.37 x 10^9
		B	2.55 x 10^9
		C	2.37 x 10^9
	2	A	2.36 x 10^9
		B	2.59 x 10^9
		C	2.36 x 10^9
	3	A	2.31 x 10^9
		B	2.95 x 10^9
		C	2.31 x 10^9
Jurkat + retronectin	1	A	6.49 x 10^9
		B	7.65 x 10^9
		C	6.49 x 10^9
	2	A	6.66 x 10^9
		B	9.28 x 10^9
		C	7.89 x 10^9
	3	A	6.51 x 10^9
		B	7.98 x 10^9
		C	1.02 x 10^{10}
T-cells	1	A	2.12 x 10^7
		B	2.02 x 10^7
		C	2.03 x 10^7
	2	A	1.43 x 10^7
		B	1.33 x 10^7
		C	1.35 x 10^7
	3	A	1.58 x 10^7
		B	1.48 x 10^7
		C	1.50 x 10^7
T-cells + retronectin	1	A	2.57 x 10^7
		B	2.43 x 10^7
		C	2.46 x 10^7
	2	A	2.22 x 10^7
		B	2.11 x 10^7
		C	2.13 x 10^7
	3	A	2.34 x 10^7
		B	2.24 x 10^7
		C	2.26 x 10^7

TABLE S2-Summary of model adequacy metrics, including R^2, RMSE, and ANOVA p-values, used to assess the performance of the DoE models.

Response	Significant Factors	RMSE	R^2	ANOVA p-value
Viability (%)	Donor, Lentiboost, Polybrene and Centrifugation Speed	0.762	0.984	< 0.0001
CAR%	Lentiboost, Polybrene, Centrifugation Speed	4.170	0.963	< 0.0001
CD4:CD8 Ratio	Donor, Lentiboost	0.415	0.901	< 0.001
Doubling Time	Donor	0.088	0.912	< 0.001

TABLE S3-List of factors, variables units and levels used in the design of experiment studies used in this manuscript.

<table>
<tr><th>Variable List</th><th>Factor</th><th>Unit</th><th colspan="2">Levels</th></tr>
<tr><td rowspan="4">Independent Variables</td><td>T cell Donor</td><td>Donor Number</td><td colspan="2">1, 2, 3</td></tr>
<tr><td>LentiBOOST™</td><td>mg.mL^{-1}</td><td>0</td><td>1</td></tr>
<tr><td>Polybrene</td><td>mg.mL^{-1}</td><td>0</td><td>0.005</td></tr>
<tr><td>Centrifugation Speed</td><td>g</td><td>1000</td><td>2000</td></tr>
<tr><td rowspan="4">Dependent Variables</td><td>CAR Expression</td><td>%</td><td colspan="2">-</td></tr>
<tr><td>CD4:CD8 Ratio</td><td>No units</td><td colspan="2">-</td></tr>
<tr><td>Doubling Time</td><td>hours</td><td colspan="2">-</td></tr>
<tr><td>Viability</td><td>%</td><td colspan="2">-</td></tr>
</table>
